# Fatty acid synthase (FASN) is a tumor-cell-intrinsic metabolic checkpoint restricting T-cell immunity

**DOI:** 10.1038/s41420-024-02184-z

**Published:** 2024-09-30

**Authors:** Elisabet Cuyàs, Stefano Pedarra, Sara Verdura, Miguel Angel Pardo, Roderic Espin Garcia, Eila Serrano-Hervás, Àngela Llop-Hernández, Eduard Teixidor, Joaquim Bosch-Barrera, Eugeni López-Bonet, Begoña Martin-Castillo, Ruth Lupu, Miguel Angel Pujana, Josep Sardanyès, Tomás Alarcón, Javier A. Menendez

**Affiliations:** 1 Program Against Cancer Therapeutic Resistance (ProCURE), Catalan Institute of Oncology, 17007 Girona, Spain; 2 Metabolism and Cancer Group, Girona Biomedical Research Institute (IDIBGI), 17190 Girona, Spain; 3 Centre de Recerca Matemàtica (CRM), 08193 Bellaterra, Barcelona Spain; 4 ProCURE, Catalan Institute of Oncology, Oncobell, Bellvitge Institute for Biomedical Research (IDIBELL), 08908 L’Hospitalet de Llobregat, Barcelona, Spain; 5 Medical Oncology, Catalan Institute of Oncology, 17007 Girona, Spain; 6 Precision Oncology Group (OncoGir-Pro), Girona Biomedical Research Institute (IDIBGI), 17190 Girona, Spain; 7 Department of Medical Sciences, Medical School, University of Girona, 17071 Girona, Spain; 8 Department of Anatomical Pathology, Dr. Josep Trueta Hospital of Girona, 17007 Girona, Spain; 9 Unit of Clinical Research, Catalan Institute of Oncology, 17007 Girona, Spain; 10 Division of Experimental Pathology, Department of Laboratory Medicine and Pathology, Mayo Clinic, Rochester, MN 55905 USA; 11 Mayo Clinic Cancer Center, Rochester, MN 55905 USA; 12 Department of Biochemistry and Molecular Biology Laboratory, Mayo Clinic Laboratory, Rochester, MN 55905 USA; 13 ICREA, 08010 Barcelona, Spain; 14 Departament de Matemàtiques, Universitat Autònoma de Barcelona, 08193 Bellaterra, Barcelona Spain

**Keywords:** Cancer metabolism, Oncogenesis

## Abstract

Fatty acid synthase (FASN)-catalyzed endogenous lipogenesis is a hallmark of cancer metabolism. However, whether FASN is an intrinsic mechanism of tumor cell defense against T cell immunity remains unexplored. To test this hypothesis, here we combined bioinformatic analysis of the FASN-related immune cell landscape, real-time assessment of cell-based immunotherapy efficacy in CRISPR/Cas9-based FASN gene knockout (*FASN KO*) cell models, and mathematical and mechanistic evaluation of FASN-driven immunoresistance. *FASN* expression negatively correlates with infiltrating immune cells associated with cancer suppression, cytolytic activity signatures, and HLA-I expression. Cancer cells engineered to carry a loss-of-function mutation in *FASN* exhibit an enhanced cytolytic response and an accelerated extinction kinetics upon interaction with cytokine-activated T cells. Depletion of *FASN* results in reduced carrying capacity, accompanied by the suppression of mitochondrial OXPHOS and strong downregulation of electron transport chain complexes. Targeted *FASN* depletion primes cancer cells for mitochondrial apoptosis as it synergizes with BCL-2/BCL-X_L_-targeting BH3 mimetics to render cancer cells more susceptible to T-cell-mediated killing. FASN depletion prevents adaptive induction of PD-L1 in response to interferon-gamma and reduces constitutive overexpression of PD-L1 by abolishing PD-L1 post-translational palmitoylation. FASN is a novel tumor cell-intrinsic metabolic checkpoint that restricts T cell immunity and may be exploited to improve the efficacy of T cell-based immunotherapy.

## Introduction

Reactivation of de novo lipogenesis catalyzed by fatty acid synthase (FASN) is a nearly universal, targetable hallmark of the metabolic remodeling that occurs in cancer cells [1–6]. The contribution of FASN to tumor initiation, cancer cell growth and survival, therapeutic resistance, and tissue-specific metastasis has received considerable attention over the past two decades [7–12]. Interestingly, we are beginning to accumulate evidence that FASN signaling may also mechanistically link cancer cell-intrinsic metabolic reprogramming to the cancer cell-extrinsic immune system.

During initial tumorigenesis, FASN is a key component of the immunoediting metabolic program that supports tumor immune evasion [13]. Exposure to interferon-γ (IFNγ) during T-cell-mediated immune surveillance leads to an “immunometabolic editing”, whereby tumor cells coordinate the simultaneous activation of FASN and immune checkpoints such as programmed death ligand-1 (PD-L1) to maximize tumor proliferation and immune evasion. The FASN lipogenic activity acquired by cancer cells to evade immune surveillance should be maintained in established tumors to limit CD8^+^ T lymphocyte infiltration and promote T cell dysfunction [13]. Similarly, FASN-mediated major histocompatibility complex class II (MHC-II) suppression is a potentially widespread mechanism underlying the cancer cell escape from early immune detection [14, 15]. The MHC-dependent antigen processing and presentation is central to promoting immune recognition of cancer cells to increase the tumor infiltration of T cells and enhance anti-cancer immunity. The accumulation of FAs produced by hyperactivated FASN is sufficient to transcriptionally silence MCH-II expression, a causal effector in cancer immune evasion [15]. Tumor FASN expression has been shown to be part of an immune-related signature that informs an immunosuppressive TME characteristic of immune-excluded tumors that may benefit from certain types of immune checkpoint inhibitors (ICIs) [16]. FASN mutations that inactivate FASN lipogenic function result in a favorable immune TME and appear to correlate with an improved response to ICIs [17]. Taken together, these findings highlight the therapeutic potential of targeting FASN to reverse the immunosuppressive features of FASN-driven cold tumors to a hot-like, immunostimulatory context. Accordingly, FASN blockade has been proposed to help overcome immune resistance and enable new therapeutic strategies to optimize current immunotherapeutics in multiple ways [18].

Here, we aimed to clarify whether FASN is a metabolic driver of immune resistance in cancer cells. We first performed a bioinformatic analysis of data from The Cancer Genome Atlas (TCGA) to determine the involvement of FASN in the immune cell landscape of human tumors. We then integrated CRISPR/Cas9-based knockout of the *FASN* gene with impedance-based cytotoxic assays to assess in real time assessment of how loss of FASN metabolic signaling alters cancer cell response to cytokine-activated T cells. The FASN dependence of the cancer cell immune escape machinery was formally defined through mathematical modeling and mechanistic exploration of the experimental data. Specifically, we examined how FASN-regulated mitochondrial OXPHOS function and priming status might affect the susceptibility of cancer cells to T cell-mediated killing [19, 20]. We then investigated whether FASN could regulate the adaptive and constitutive expression of the immune checkpoint PD-L1 in cancer cells, including FASN-driven PD-L1 palmitoylation, a critical post-translational modification required for the structural integrity and functionality of cell membrane-bound PD-L1 [21, 22]. Through such a multifaceted approach, we now provide evidence that FASN is a tumor cell-intrinsic metabolic determinant that disfavors immune-mediated cytolysis. This supports the notion that FASN would represent a potential immunotherapeutic target to enhance T cell antitumor efficacy.

## Results

### *FASN* gene expression negatively correlates with immune escape landscapes

*FASN* expression measured by RNA sequencing (RNA-seq) in The Cancer Genome Atlas (TCGA) [23] was considered to be biologically relevant as it was found to positively correlate with curated gene signatures of lipid metabolism and AMPK signaling [24] in both the breast cancer and pan-cancer datasets (Pearson correlation coefficient [PCC] = 0.28–0.30, *p*-values < 10^−^^21^; Fig. S2). In addition, *FASN* expression was positively correlated with a signature corresponding to the top 100 CRISPR-mediated gene dependencies similar to *FASN* identified in the Cancer Cell Line Encyclopedia (breast cancer TCGA PCC = 0.30, *p* = 4 × 10^−^^24^; pan-cancer TCGA PCC = 0.75, *p* < 10^−^^16^; Fig. S3) [25].

**Fig. 1:**
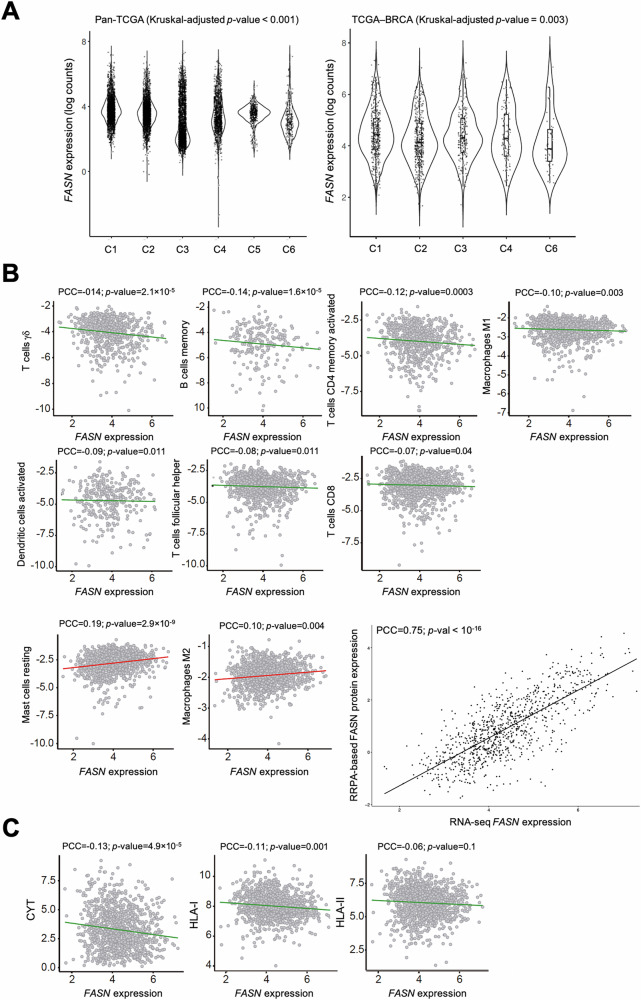
Correlation of *FASN* expression with inferred immune cell types and characteristics. **A** *FASN* expression distribution across the C1-C6 TCGA transcriptomic immune subtypes: C1 (wound healing), C2 (IFNγ dominant), C3 (inflammatory), C4 (lymphocyte depleted), C5 (immunologically quiet), and C6 (TGFβ dominant). Kruskal-Wallis-adjusted *p*-values are shown. **B** Negative (*upper* and *middle panels*) and positive (*lower panels*) correlations of *FASN* (gene expression, log_2_) with CIBERSORTx-inferred immune cell content (Y-axis) in TCGA primary breast tumors. The inset shows the correlation between FASN gene expression by RNA-seq and FASN protein expression by RPPA. The PCCs are indicated (FDR-adjusted *p*-values < 0.05). **C** Negative correlation of *FASN* (gene expression, log_2_) with inferred cytolytic activity (CYT) and gene expression signatures of HLA-I and HLA-II components. PCCs are indicated (FDR-adjusted *p*-values < 0.05).

*FASN* gene expression was then analyzed in relation to the TCGA global immune classification of solid tumors, which established six major (C1-C6) transcriptomic immune subtypes [26] (Fig. 1A). *FASN* was found to be overexpressed in tumors classified as immune subtype C1, defined as “wound healing”, which exhibited elevated expression of angiogenic genes, a high proliferation rate, and a Th2 cell bias toward the adaptive immune infiltrate. In contrast, *FASN* was found to be underexpressed in the immune subtype C6, defined as “TGFβ dominant”, which comprises a small group of mixed tumors that have the highest TGFβ signature (p < 10^−34^) and a high lymphocytic infiltrate with an even distribution of type I and type II T cells [26].

To assess the relationship between *FASN* gene expression and immune status in breast tumors (TCGA BRCA), we inferred immune cell content by applying CIBERSORTx to the bulk RNA-seq profiles [27, 28] (Fig. 1B). *FASN* expression was negatively correlated (PCCs < 0, false discovery rate [FDR]-adjusted *p* values < 0.05) with immune cell types thought to be associated with cancer suppression, including γδ T cells, B memory cells, activated CD4 T memory cells, M1 macrophages, activated dendritic cells, follicular helper T cells, and CD8 T cells. In turn, *FASN* expression was found to be positively correlated (PCCs > 0, FDR-adjusted *p*-values < 0.05), with two immune cell types generally associated with immune evasion, resting mast cells and M2 macrophages (Table S1). *FASN* gene and protein expression (as measured by reverse phase protein array [RPPA] assays in the TCGA [29]) also showed a robust positive correlation (PCC = 0.75, *p* < 10^−^^16^), further supporting the biological relevance of *FASN* expression measurements (Fig. 1B). Violin plots of the tertile stratification of *FASN* expression confirmed the relationships between FASN and the content of cells involved in immune evasion (Fig. S4). A multivariate regression analysis including data from 13 TCGA studies across multiple tumor types confirmed all of the above negative correlations between *FASN* expression and immune cell landscapes in breast cancer (β estimate < 0, *p* < 0.01), but failed to detect any positive significant associations (Table S2; Fig. S3).

Consistent with the CIBERSORTx-based predictions, *FASN* expression was found to be negatively correlated with a signature of cytolytic activity (CYT) and HLA-I expression in breast tumors [30] (Fig. 1C). TCGA pan-cancer analyses showed a robust negative correlation between *FASN* expression and cytolytic activity, HLA-I, and HLA-II immune signatures (PCCs = −0.22, −0.23, −0.26, FDR-adjusted *p*-values < 10^−^^100^). In a multivariate regression analysis, the negative association between FASN expression and cytolytic activity and HLA-II remained significant (*p* < 10^−^^7^), and the association with HLA-I showed a similar trend (*p* = 0.057). Thus, across cancer types, high *FASN* expression is often associated with tissue, cellular, and molecular features of immune evasion.

### *FASN* gene expression positively correlates with hallmarks of immune escape

**Fig. 2:**
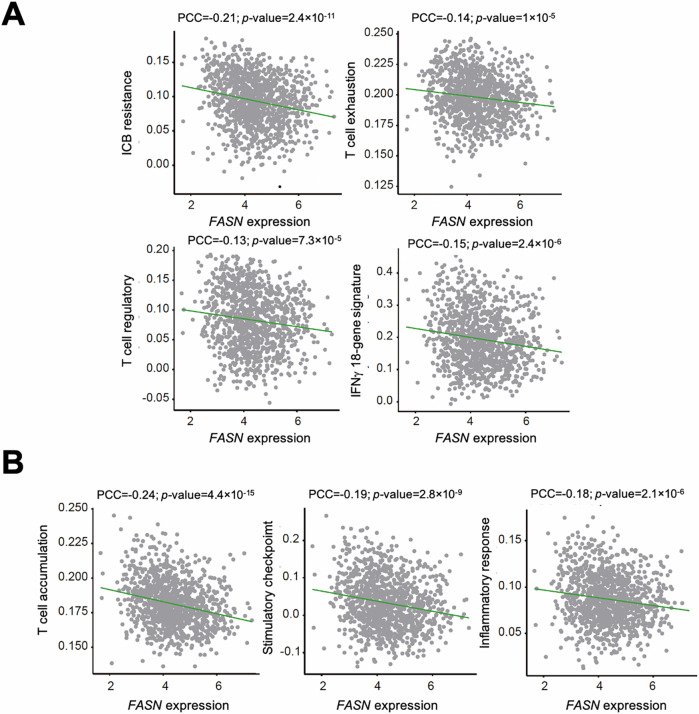
Correlation of *FASN* expression with immune system and immunotherapy-related signatures. **A** Negative correlation of *FASN* (gene expression, log_2_) immunotherapy-related gene signatures, including acquired resistance (*top left*), CD8 T-cell exhaustion (*top right*), activated regulatory T cells (*bottom left*), and IFNγ-mediated prediction of response to anti-PD1 therapy (*bottom right*). PCCs are indicated (FDR-adjusted *p*-values < 0.05). **B** Negative correlation of *FASN* (gene expression, log_2_) with signatures of T cell accumulation in tumors (*left*), genes encoding for stimulatory checkpoints (*middle*), and biological processes of the inflammatory response (*right*). PCCs are indicated (FDR-adjusted *p*-values < 0.05).

To further investigate the predicted relationship between *FASN* expression and cancer immune evasion, we analyzed signatures associated with response or resistance to immunotherapy [31, 32]. *FASN* expression was negatively correlated with gene sets defining activated regulatory T cells, IFNγ-mediated prediction of response to anti-PD1 therapy, exhausted CD8 T cells, and acquired immunotherapy resistance (PCCs ≤ −0.13, FDR-adjusted *p*-values < 0.05) [33–35] (Fig. 2A). *FASN* expression also anticorrelated with a signature of T-cell accumulation in tumors [36], which was consistent with gene sets involved in stimulatory immune checkpoints and inflammatory response [26] (Fig. 2B). The relationships between FASN status and immunotherapy-related resistance signatures were confirmed by tertile stratification of *FASN* expression (Fig. S4). Extending these observations, *FASN* expression negatively correlated with three derived measures of T-cell receptor (TCR) repertoire enrichment in both breast cancer and pan-cancer datasets (PCCs ranging from −0.014 to −0.06; FDR-adjusted *p*-values < 10^−^^4^). Taken together, these analyses suggest that *FASN* gene expression is negatively correlated with cancer-suppressing immune landscapes and positively correlated with features of cancer immune escape.

### *FASN* loss sensitizes cancer cells to cytokine-activated T-cells (CATs)

To evaluate the impact of FASN-driven de novo FA synthesis on tumor cell responses to T cells, we performed impedance-based real-time monitoring of the cytolytic response to cell-based immunotherapy in co-isogenic cell lines that were either wild-type or deficient in the *FASN* gene (Fig. S1). We chose the chronic myeloid leukemia (CML)-derived, near-haploid cell line HAP1, in which CRISPR/Cas9-mediated knockdown of the *FASN* gene has been previously established as a suitable model system for defective de novo FA synthesis [37, 38]. This was confirmed by a significant increase in the FASN substrate malonyl-CoA and a striking resistance to targeted FASN inhibitors in *FASN KO* HAP1 cells [37, 38]. As cell-based immunotherapy we choose cytokine-activated T-cells (CATs) cells, which are a heterogenous population of effector T cells generated from peripheral blood mononucleated cells (PBMCs) cultured under cytokine stimulation, possessing non-MHC-restricted cytolytic activity against tumor cells [39–44].

**Fig. 3:**
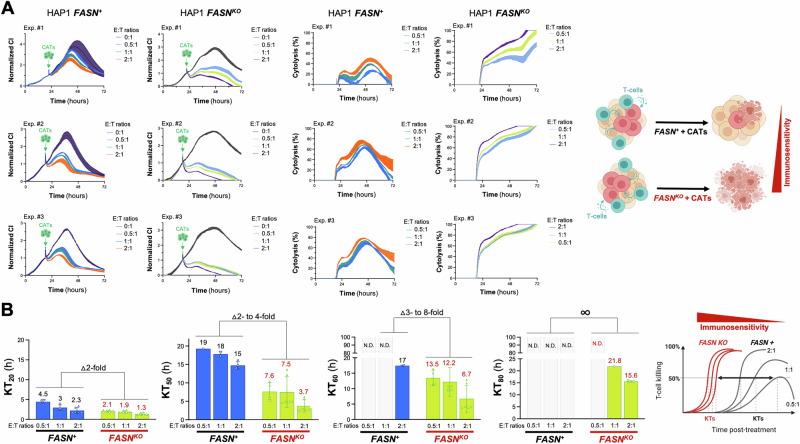
Effect of *FASN* loss on the sensitivity of HAP1 cancer cells to cytolysis by cytokine-activated T cells (CATs). **A**. *Left*. Raw CI plots (*n* = *3* independent experiments) of parental *FASN*^*+*^ HAP1 and *FASN*^*KO*^ HAP1 derivatives (target cells) incubated with different E:T ratios of CATs. Y-axis is the normalized CI (for the CI value measured before the addition of CATs) generated by the RTCA software and displayed in real time. X-axis is the cell culture/treatment time in hours (up to 72 hours). The spike in the CI signal is noise due to the removal of the E-plates from the incubator to add CATs. *Right*. CI plots were converted to % cytolysis plots using the xCELLigence Immunotherapy Software (xIMT) after addition of CATs at different E:T ratios. **B** Killing kinetics of CATs against HAP1-*FASN*^*+*^ and HAP1-*FASN*^*KO*^ derivatives using 20%, 50%, 60%, and 80% Killing Time (KT) values calculated with the xIMT software from the impedance measurements shown in (**A**). Figure shows means (*columns*) of KT values ± S.D. (*bars*). ND Not detected. In the absence of *FASN*, there is a marked leftward shift in the KT values at any E:T ratio, indicating increased immunosensitivity.

Using the xCELLigence instrument (Fig. S1), we assessed cytolysis of target (T) HAP1 cancer cells as a function of exposure time to increasing numbers of effector (E) CATs to apply an increasing selection pressure. CRISPR-mutagenized *FASN*^*KO*^ HAP1 cells and *FASN*^*+*^ HAP1 parental counterparts were seeded in E-plates and treated the next day with CATs at 0.5:1, 1:1, and 2:1 E:T ratios. As recommended by the manufacturer, the target cell index (CI) was 0.5–1 and in the linear range prior to exposure to CATs. Both the extent of CI decrease and the percentage of cytolysis varied drastically between *FASN*^*+*^ HAP1 parental cells and *FASN*^*KO*^ HAP1 derivatives exposed to low (0.5:1)– to –high (2:1) E:T ratios of CATs (Fig. 3A). Although the addition of CATs resulted in a less pronounced slope of the RTCA profiles in *FASN*^*+*^ HAP1 parental cells, none of the effector ratios was able to reduce the CI signal of target cells to background levels. Indeed, all the CATs-treated *FASN*^*+*^ HAP1 populations reached a long-term equilibrium at CIs between 0.5 and 1 that may reflect the limited capacity of CATs to kill all the target *FASN*^*+*^ cancer cells regardless of the E:T ratio employed. A completely different picture emerged when monitoring the dynamics of CATs-mediated cytolysis in *FASN*^*KO*^ HAP1 isogenic derivatives. The addition of CATs to *FASN*^*KO*^ HAP1 cells was followed by an immediate and time-dependent reduction in normalized CI values (Fig. 3A), which quickly reached background levels, consistent with a complete cytolysis of the target *FASN*^*KO*^ HAP1 cancer cells.

The fact that the time dependence of cytolysis is captured over multiple measurements allows the derivation of kinetic parameters such as the so-called “killing time” (KT), i.e., the time required to reach a % cytolysis (e.g., 20%, 40%, 50%, 80%) at a given E:T ratio. The KT parameter allows a careful analysis in the time dimension, ranking the treatments according to the rate of cell killing. As such, the KT parameter is complementary to the % cytolysis parameter, which shows the efficacy of a given condition at a given time. At low to intermediate percentages of cytolysis (20% to 50%), CRISPR-mutagenized *FASN*^*KO*^ HAP1 cells exhibited an up to 8-fold faster killing kinetics compared to parental *FASN*^*+*^ HAP1 cells (Fig. 3B). Crucially, most if not all of the E:T ratios failed to achieve higher percentages (60% to 80% or greater) within the time frame of the assay in *FASN*^*+*^ HAP1 cells, and therefore KT_80_ values could not be determined for this condition. Conversely, physiologically relevant E:T ratios of 0.5:1 and 1:1 were still able to achieve 60-80% cytolysis in *FASN*^*KO*^ HAP1 cells (Fig. 3B). Taken together, these results demonstrate that the suppression of *FASN* allows CATs to kill cancer cells with a significantly higher degree of efficiency.

### *FASN* loss reduces metabolic fitness and renders cancer cells more susceptible to killing by cytokine-activated T cells

To test whether the FASN-driven susceptibility of HAP1 cancer cells to cytolysis by CATs is related to changes in either cell growth rate or fitness landscapes, raw experimental data from the real-time cell analyzer were used to estimate parameters of mathematical models aimed at delineating growth and killing dynamics between cancer cells and CATs. We applied macroevolutionary algorithms, heuristic optimization methods that are capable of simulating the extinction patterns in a network ecosystem where the dynamics are based only on the relationship between “species” [45, 46] –e.g., cancer cells and cytotoxic T cells (i.e., CATs).

**Fig. 4:**
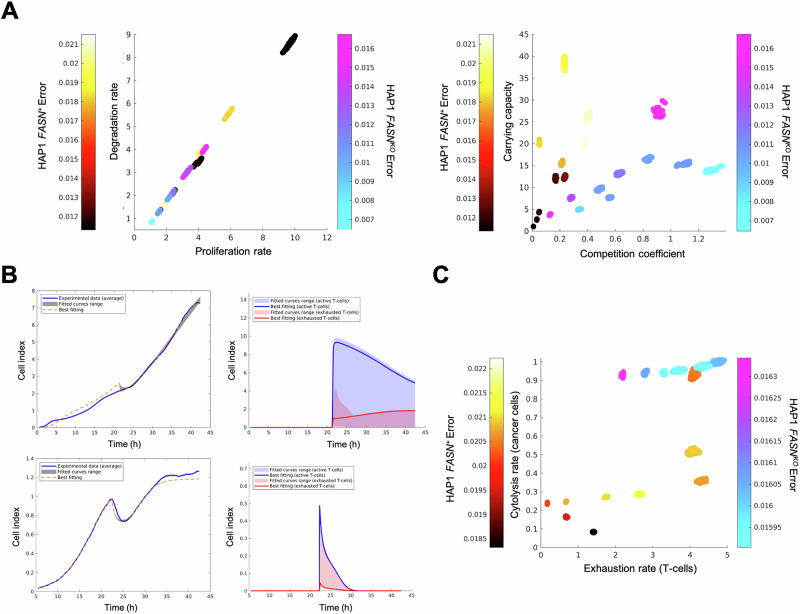
Mathematical deconvolution of T-cell killing and exhaustion from real-time killing assay data. **A** Comparison of intrinsic parameters of cancer cell models (*FASN*^*+*^ HAP1 parental cells in hot colors and *FASN*^*KO*^ HAP1 derivatives in cold colors). *Left*. Scatter plot showing the comparison between growth (*r*) and death (*d*) rates. The growth rate (i.e., *r-d*) is the same for both cell types. *Right*. Scatter plot showing the comparison between the weight (alpha_1_) and the carrying capacity (K). **B** Experimental (blue curve) and fitted cell index data of *FASN*^*+*^ HAP1 parental cells (*top*) and *FASN*^*KO*^ HAP1 derivatives (*bottom*) growing in the presence of CATs. *Left panels*. Fitted cancer cell index data. *Right panels*. Dynamics for the active and exhausted T cells obtained from the model for the parameter values that provide the best fits to the experimental data. The parameters for best fits are: for HAP1 *FASN*^*+*^, *r* = (2.701; 2.342; 4.462), *d* = (2.141; 1.757; 3.834), *a* = (0.834; 0.036; 0.677), *K* = 1.794, *h* = (0.642; 0.356; 0.084), *l* = (3.845; 3.403; 1.437), *b* = (0.548; 0.378), *r*_A_ = 23.300, *d*_E_ = 29.383, *C* = 6.334; for HAP1 *FASN*^*KO*^, *r* = (4.424; 6.162; 10.005), *d* = (3.975; 5.922; 9.659), *a* = (0.175; 0.980; 0.138), *K* = 2.037, *h* = (0.955; 4.212; 3.677), *l* = (3.875; 1.637; 1.597), *b* = (0.169; 0.469), *r*_A_ = 2.671, *d*_E_ = 35.676, *C* = 72.179. The parameter values shown in triplicate correspond to the three tumor cell populations. **C** Comparison of the interaction parameters *I* (exhaustion) and *h* (cytolysis). *FASN*^*+*^ HAP1 parental cells are shown in hot colors and *FASN*^*KO*^ HAP1 derivatives are shown in cold colors. The bars with the color gradients show the error, calculated as the average absolute value of the difference between the fitted data and the experimental data at each time step.

We first analyzed the population dynamics of parental *FASN*^*+*^ HAP1 versus isogenic *FASN*^*KO*^ HAP1 cells. After multiple iterations of macroevolutionary time course fitting for both cell populations, we confirmed that *FASN* elimination has a significant negative impact on both CI –a parameter that reflects the state of cell growth, proliferation, cell size, cell-to-cell contact and cell-substrate attachment– and the fitness landscape of cancer cells in terms of their ability to utilize resources (Fig. S5, Fig. 4A). Specifically, the carrying capacity of HAP1 cells –the maximum population size that can be supported by available resources and phenotypic characteristics of individual cells [47–49]– was significantly reduced upon mutational elimination of the *FASN* gene (Fig. 4A). Differences in the growth time courses of *FASN*^*+*^ and *FASN*^*KO*^ cell populations cannot be explained by the effect of FASN on cell division rates, but rather by the fine-tuning of the interaction between resource availability and metabolic rates of cancer cells (Fig. 4B). In this context, mathematical modeling confirmed that targeted *FASN* gene elimination in cancer cells leads to an accelerated extinction kinetics upon interaction with T cells, as shown by the increased cytolytic rates associated with the interaction between *FASN*^*KO*^ cells and T cells. As expected, higher cytolysis rates are correlated with higher rates of exhausted T cells (Fig. 4C). Our biomathematical model suggests that suppression of FASN-driven lipogenesis renders cancer cells less efficient in the use of metabolic resources and more susceptible to killing by cytotoxic T lymphocytes.

### *FASN* loss suppresses mitochondrial respiration

**Fig. 5:**
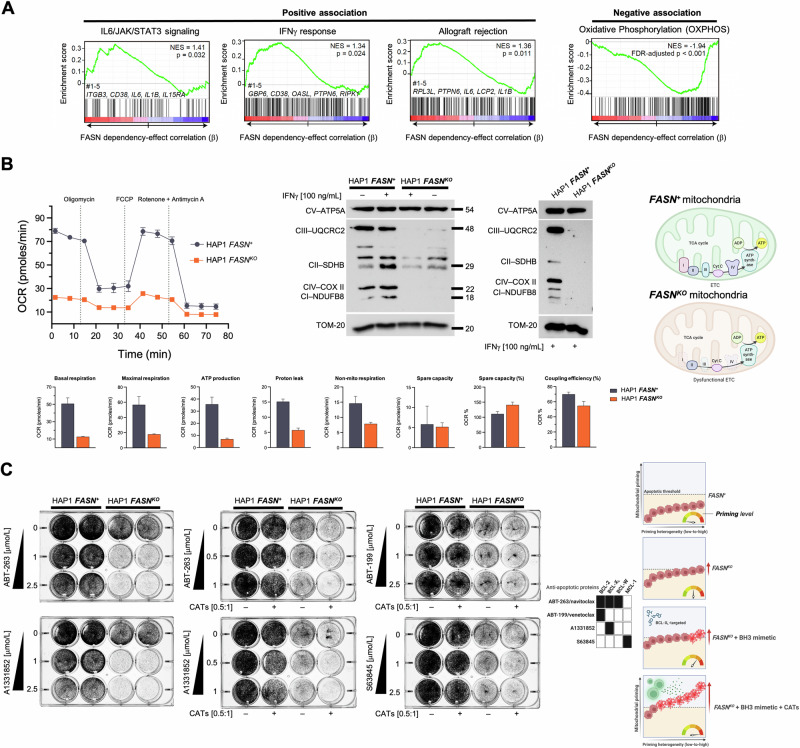
Effect of *FASN* loss on mitochondrial OXPHOS function and priming. **A** GSEA results for the positive and (strongest) negative association between FASN and immune system-related gene sets in the CRISPR-based dependencies of the CCLE. The GSEA normalized enrichment score (NES) and nominal *p*-values, as well as the top 5 associated genes, are shown for each positively associated feature. The GSEA NES and FDR-adjusted *p*-value are shown in the negatively associated feature. **B** *Left*. Mitochondrial function in *FASN*^*+*^ HAP1 parental cells and *FASN*^*KO*^ HAP1 derivatives was assessed using the Seahorse XFp Cell Mito Stress Test Assay. The figure shows representative Seahorse OCR bioenergetic profiles (*n* ≥ 3) that were acquired after sequential addition of pharmacological inhibitors to examine the function of individual components of the mitochondrial electron transport chain (ETC). To estimate the fraction of basal OCR coupled to ATP synthesis, ATP synthase (ETC complex V) was inhibited by oligomycin, which reduces the OCR rate to the extent that cells use mitochondria to generate ATP. Proton leak across the mitochondrial membrane is responsible for the remaining OCR. The proton ionophore FCCP was injected for determination of the maximum OCR that the cells could sustain. Finally, antimycin A was injected to inhibit the flow of electrons through the ETC complex III, which leads to a dramatic suppression of the OCR. The remaining OCR is attributable to O_2_ consumption due to formation of mitochondrial ROS and non-mitochondrial sources. Spare capacity is calculated as the maximal rate minus the basal rate and represents a parameter the cells can use to back up increased work to cope with stress. *Right*. Expression of mitochondrial ETC proteins in *FASN*^*+*^ HAP1 parental cells and *FASN*^*KO*^ HAP1 derivatives. Representative immunoblot of ETC complex I (NDUFB8), complex II (SDHB), III (UQCRC2), IV (COXII) and V (V-ATP5A) protein expression proteins in *FASN*^*+*^ HAP1 parental cells and *FASN*^*KO*^ HAP1 derivatives cultured in the absence or presence of IFNγ (100 nmol/L) for 48 h. Similar results were obtained from two additional independent experiments. In response to targeted *FASN* loss of function, mitochondrial OXPHOS dysfunction occurs with drastic downregulation of ETC complexes I, III and IV. **C** *FASN*^*+*^ HAP1 parental cells and *FASN*^*KO*^ HAP1 derivatives (60,000 cells/well) were challenged with increasing concentrations of BH3 mimetics (ABT-263/navitoclax, A1331852, ABT-199/venetoclax, S63845) in the absence or presence of CAT cells at a fixed E:T ratio of 0.5:1. Tumor cells were fixed and stained and subjected to crystal violet staining 2 days later. Representative microphotographs (*n* = 3) are shown. *FASN* loss-of-function appears to enhance the mitochondrial apoptotic priming state to bring tumor cells closer to the apoptotic threshold, a phenomenon that can be further enhanced by pro-apoptotic, BCL-X_L_-targeting BH3 mimetics, thereby facilitating the cytolytic activity of immune cells.

To identify cancer cell dependencies similar to the consequence of depleting *FASN* expression that may underlie the reduced metabolic fitness and increased susceptibility to killing by cytotoxic T cells, we analyzed CRISPR-based gene effects identified in the Cancer Cell Line Encyclopedia (CCLE). A multivariate regression analysis evaluated the association between *FASN* knockout (KO) and any other gene *KO* effect in >1,000 cancer cell lines. Gene set-based analysis of the estimated gene associations (β) with *FASN* KO showed the strongest (nominal *p* < 0.05) positive correlations with three hallmarks of the immune system, namely IL6-JAK-STAT3 signaling, IFNγ response, and allograft rejection (Fig. 5A). The top 100 *FASN* positive codependences were found to be enriched (FDR-adjusted *p*-value < 0.05) in the Gene Ontology term “*cytokine receptor binding*” (identified genes included *DAB2IP*, *IL5*, *IL36B*, *ITGB3*, *GH2*, *TFF2*, and *TLR5*). The strongest negative association corresponded to the features “*adipogenesis*” (nominal *p* = 0.025) and “*oxidative phosphorylation (OXPHOS)*” (FDR-adjusted *p* < 0.001; Fig. 5A).

To experimentally validate that loss of FASN metabolic signaling may be associated with changes in the mitochondrial respiration of cancer cells, the oxygen consumption rate (OCR) of *FASN*^*KO*^ HAP1 cells was compared to that of *FASN*^*+*^ HAP1 parental cells using the Seahorse XF Extracellular Flux Analyzer. *FASN KO* HAP1 cells exhibited drastically reduced mitochondrial OXPHOS and ATP production, with significantly lower basal and maximal respiratory capacities compared to *FASN*^*+*^ HAP1 parental cells (Fig. 5B).

Central to mitochondria respiration is the activity of distinct electron transport chain (ETC) complexes. To investigate whether the observed effects on the bioenergetic functions of *FASN*^*KO*^ HAP1 cells were associated with changes in the protein abundance of five different OXPHOS systems, we used an antibody cocktail to examine whether FASN suppression was associated with corresponding changes in the protein abundance of ETC complex I (nuclear-encoded NDUFB8), complex II (nuclear-encoded SDHB), III (nuclear-encoded UQCRC2), IV (mitochondrial-encoded COXII), and V (nuclear-encoded V-ATP5A). Strikingly, complex I, III, and IV were almost completely absent in *FASN KO* HAP1 cells (Fig. 5B). In addition, while the abundance of CII was relatively unaffected, its expression was downregulated in response to IFNγ in *FASN* KO cells, but upregulated in the parental *FASN*^*+*^ HAP1 counterparts. Complex V was completely unaffected by the loss of *FASN*.

Loss of *FASN* results in a severe mitochondrial respiratory deficiency, characterized by a striking loss of ETC-OXPHOS complexes, which may account for the reduced carrying capacity (and increased susceptibility to T-cell cytolysis) of *FASN*^*KO*^ cancer cells.

### Elimination of FASN increases susceptibility of cancer cells to CAT killing by lowering the mitochondrial apoptotic threshold

We have previously reported that FASN inhibition can result in increased mitochondrial apoptosis priming, placing cancer cells in a primed-for-death state that is “addicted” to BCL-2 anti-apoptotic proteins [20]. When cancer cells are primed and pushed closer to apoptotic thresholds, less potent hits from cytolytic immune cells or less frequent hits due to low E:T ratios may be sufficient to push cancer cells over mitochondrial apoptotic thresholds [19]. Because loss of OXPHOS ETC increases BCL-2 dependence and the primed state of cancer cells [50], we explored whether mitochondria might operate as mechanistic amplifiers of the death signal delivered by cytolytic immune cells in *FASN*^*KO*^ cells [19]. We tested whether co-treatment with BH3 mimetics –small molecules that block the interaction of specific proapoptotic factors with cognate antiapoptotic BCL-2 family proteins, thereby releasing bound proapoptotic activators [51–54]– could render *FASN*^*KO*^ cell populations easier targets for CATs. *FASN*^*KO*^ cell populations were more sensitive to the pan-BCL2 inhibitor ABT-263/navitoclax and the BCL-X_L_-specific inhibitor A1331852 than *FASN*^*+*^ cell populations (Fig. 5C). Furthermore, ABT-263/navitoclax and A1331852, but not BCL2-specific ABT-199/venetoclax or MCL-1-specific S63845, synergized with physiologically relevant low E:T ratios (0.5:1) of CATs in killing *FASN*^*KO*^ but not *FASN*^*+*^ cells.

Taken together, these results suggest that suppression of FASN increases the susceptibility of cancer cells to CAT killing through a mechanism that may involve alterations in mitochondrial priming (Fig. 5C).

### FASN loss targets adaptive and constitutive up-regulation of PD-L1 via post-translational palmitoylation

**Fig. 6:**
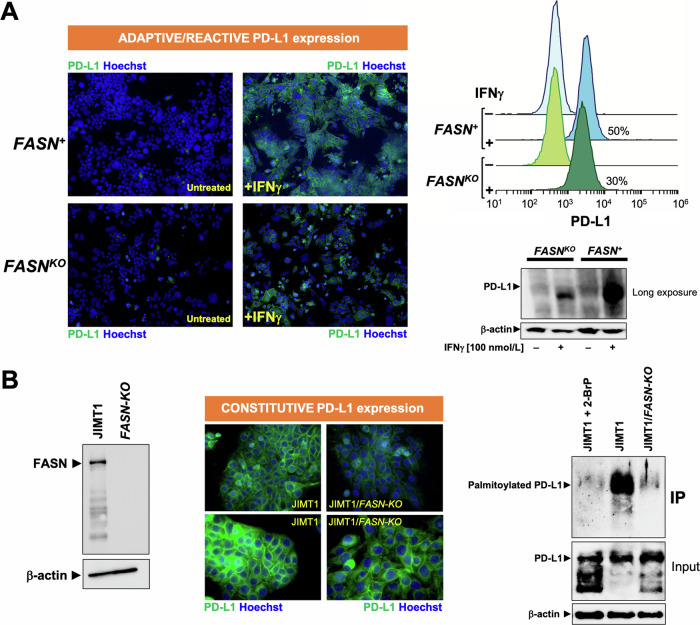
Effect of *FASN* loss on adaptive/reactive and constitutive PD-L1 expression. **A** *Left*. Representative immunofluorescence staining of PD-L1 in *FASN*^*+*^ HAP1 parental cells and *FASN*^*KO*^ HAP1 derivatives, either with or without IFNγ (100 nmol/L, 24 h) exposure. *Right*. Flow cytometry-based PD-L1 cell surface expression in *FASN*^*+*^ HAP1 parental cells and *FASN*^*KO*^ HAP1 derivatives, either with or without IFNγ (100 nmol/L, 24 h) exposure. Representative immunoblot analysis of PD-L1 expression in *FASN*^*+*^ HAP1 parental cells and *FASN*^*KO*^ HAP1 derivatives, either with or without IFNγ (100 nmol/L, 24 h). Each cell line was tested in at least three independent experiments. **B** *Left*. Representative immunoblot analysis of FASN expression in JIMT1 parental cells and JIMT1/*FASN-KO* derivatives. *Middle*. Representative immunofluorescence staining of PD-L1 in *FASN*^*+*^ JIMT1 parental cells and JIMT1/*FASN-KO* derivatives. *Right*. Representative immunoblot analysis of whole lysates or immunoprecipitates of S-palmitoylated proteins in JIMT1 parental cells (either with or without 2-bromopalmitate) and JIMT1/*FASN-KO* derivatives. Each cell line was tested in a minimum of three independent experiments.

Expression of the immune checkpoint PD-L1 on cancer cells is adaptively induced in response to various forms of cell-based immunotherapy [55, 56]. Because PD-L1-negative HAP1 cells can acquire high levels of cell surface-associated PD-L1 after exposure to IFNγ [57] –a key inflammatory cytokine secreted by T cells and NK cells that operates as the most well-known factor that induces PD-L1 in tumor cells [58–60]–, they provide an idoneous in vitro model to study whether FASN signaling is a regulator of PD-L1-driven adaptive immune resistance [61, 62]. Immunofluorescence analysis confirmed that PD-L1-negative *FASN*^*+*^ HAP1 parental cells acquired extremely high levels of cell surface PD-L1 in response to stimulation with IFNγ (Fig. 6A). CRISPR-mutagenized *FASN*^*KO*^ HAP1 cells, however, exhibited significantly attenuated PD-L1 upregulation in response to IFNγ. Flow cytometric analysis revealed a significantly lower number of PD-L1-positive cells in the IFNγ-treated *FASN*^*KO*^ HAP1 cell population as compared to the IFNγ-treated parental (*FASN*^*+*^) HAP1 counterpart (Fig. 6A). Immunoblotting analysis further confirmed that the loss of *FASN* in HAP1 cells led to a significantly reduced upregulation of PD-L1 in response to IFNγ (Fig. 6A). These findings suggest that FASN metabolic signaling contributes to the IFNγ-induced adaptive/reactive upregulation of PD-L1 in cancer cells.

Innate immune resistance involves the overexpression of immune checkpoints such as PD-L1, which is maintained by constitutively active, tumor cell-intrinsic oncogenic signaling pathways [63–66]. To investigate whether FASN signaling is involved in maintaining constitutive PD-L1 overexpression, we took advantage of JIMT-1 cells, a unique model of breast cancer with 100% of the cells being PD-L1 positive without involving an increased *PD-L1* gene copy number [67–69]. JIMT-1 cells were infected with Edit-R All-in-one lentiviral particles in which CRISPR guide FASN-sgRNA sequences and Cas9 are cloned into the same optimized lentiviral expression backbone. Immunoblotting procedures confirmed a complete loss of FASN expression in CRISPR-mutagenized *FASN KO* JIMT-1 cells (Fig. 6B). Immunofluorescence microscopy analysis showed that cell membrane-associated PD-L1 signals were clearly diminished in response to FASN loss in JIMT-1 cells, which was accompanied by a notorious redistribution of the PD-L1 staining pattern from the cell membrane to punctuate cytoplasmic vesicles (Fig. 6B).

PD-L1 palmitoylation is a post-translational modification (PTM) required for the structural integrity and functionality of cell membrane-bound PD-L1 that can be targeted to efficiently promote PD-L1 degradation and enhance T-cell immune responses against tumours [21, 22, 63, 70–73]. FASN-catalyzed de novo synthesis of palmitate provides not only a precursor for endogenous fatty acids but also the acyl group that is added to cysteine residues during post-translational palmitoylation of proteins [74–77]. Therefore, we hypothesized that the palmitoylation of PD-L1 may be impaired after KO of the *FASN* gene. Using the commercially available CAPTUREome™ S-Palmitoylated protein kit assay, a robust approach to the identification of S-palmitoylated protein species by resin-assisted capture (acyl-RAC) [78], we evaluated whether FASN could regulate PD-L1 palmitoylation. After palmitoylation enrichment, immunoblotting analyses confirmed that *FASN* gene KO was as efficient as treatment with the pan-palmitoyltransferase inhibitor 2-BrP in completely abolishing PD-L1 palmitoylation (Fig. 6C). Taken together, these results confirm that FASN loss-of-function prevents the expression of cell membrane-associated PD-L1, at least in part, by blocking the palmitoylation PTM step of PD-L1 [73, 79–81].

### FASN contributes to intrinsic cancer cell immuno-resistance in PD-L1-overexpressing/HER2-positive breast cancer cells

**Fig. 7:**
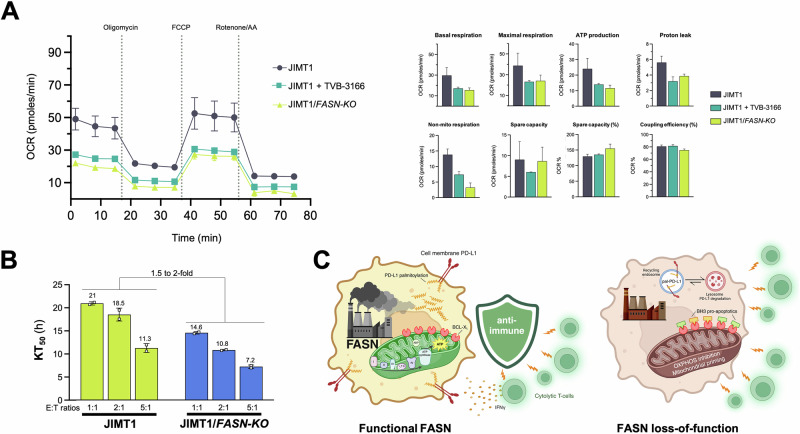
Effect of *FASN* loss on the sensitivity of PD-L1/HER2-overexpressing JIMT1 breast cancer cells to cytolysis by cytokine-activated T cells (CATs). **A** Mitochondrial function in JIMT1 parental cells and JIMT1/*FASN-KO* derivatives was assessed using the Seahorse XFp Cell Mito Stress Test Assay. The figure shows representative Seahorse OCR bioenergetic profiles (*n* ≥ 3) that were acquired after sequential addition of pharmacological inhibitors to examine the function of individual components of the mitochondrial ETC as described in Fig. 5. **B** Killing kinetics of CATs against JIMT1 parental cells and JIMT1/*FASN-KO* derivatives using 50% Killing Time (KT) values calculated with the xIMT software as described in Fig. 3. **C** FASN is a tumor-cell-intrinsic metabolic checkpoint that restricts T-cell immunity by protecting mitochondrial OXPHOS function, reducing mitochondrial priming, and promoting PD-L1 trafficking to the cancer cell membrane via PD-L1 palmitoylation (created with Biorender.com).

To investigate whether FASN might contribute to innate cancer cell resistance to cytotoxic T cells, we examined how loss of FASN impacted the response of PD-L1-overexpressing JIMT-1 cells, a model of highly aggressive trastuzumab-resistant and anti-PD-L1 poorly responder HER2 + /basal-like breast cancer [82–86], to CATs. First, we aimed to confirm whether the ability of FASN signaling to regulate mitochondrial OXPHOS was not restricted to HAP1 cells. Significantly reduced mitochondrial/non-mitochondrial respiration and ATP production in JIMT-1/*FASN*-*KO* compared to JIMT1 parental cells was confirmed by characterization of OCR profiles using the Seahorse analyzer (Fig. 7A). The small-molecule FASN inhibitor TVB-3166 [11, 12] closely mimicked the effects of targeted *FASN* gene suppression at drastically reducing mitochondrial function. Second, we used the xCELLigence system to monitor whether FASN is essential for protecting HER2+ breast cancer cells from CATs killing. Loss of *FASN* significantly decreased KT with increasing percentages of cytolysis at low E:T ratios such as 1:1 (Fig. 7B). At high E:T ratios (e.g., 5:1), only a minimal increased sensitization to CATs cytolysis was observed in *FASN*^*KO*^ JIMT-1 cells. While FASN may be dispensable at high E:T ratios, at physiologically relevant low E:T ratios, the FASN lipogenic machinery appears to be essential for protecting HER2 + /PD-L1+ breast cancer cells from elimination by T-cells.

Taken together, these results suggest that FASN may function as a tumor-cell-intrinsic marker of immune resistance that can be targeted to (re)sensitize highly aggressive cancer subtypes (e.g., HER2 + /PD-L1-overexpressing basal/HER2 breast cancer) to T-cell immunity.

## Discussion

Cancer cells are intrinsically buffered against a wide variety of metabolic and pro-apoptotic stresses, including those imposed by T cells. Here, we were interested in determining whether the key lipogenic enzyme FASN could impair immune-mediated cytolysis of tumor cells. We took advantage of cancer cells engineered to carry a loss-of-function in FASN as a unique opportunity to investigate whether cancer cells may adapt to the loss of de novo FA biosynthesis when fighting T-cell attack. We now propose that a basic metabolic program such as FASN-dependent endogenous lipogenesis is a metabolic checkpoint that functions as part of the intrinsic defense mechanisms that cancer cells employ to escape to the stress imposed by infiltrating T cells.

FASN is emerging as a key driver of the activation, differentiation, and functionality of immune cells in the tumor microenvironment (TME). FASN-mediated de novo FA synthesis is critical for the functional maturation of regulatory T cells (T_reg_ cells), which are essential for immune tolerance but also drive immunosuppression in the TME [87]. Accordingly, loss of FASN in T_reg_ cells is sufficient to inhibit tumor growth. Cancer cell-intrinsic FASN prevents anti-tumor immunity by reducing the ability of dendritic cells to maintain T cells in ovarian cancer [88] and has been associated with reduced immune infiltration in gastric cancer [89]. Here, we encountered that *FASN* expression negatively correlates with infiltrating immune cells associated with cancer suppression, cytolytic activity signatures, and HLA-I expression. *FASN* expression also anticorrelates with gene sets characteristic of immunotherapy resistance, but positively correlates with several hallmarks of immune escape, including IFNγ response. *FASN* expression also appears to correlate with response to the anti-PD-L1 monoclonal antibody atezolizumab, particularly in those tumors that co-express high levels of FASN and ICPs. Accordingly, genetic or pharmacological inhibition of FASN has been found to increase MHC-I (also called HLA-I) levels in hepatocellular carcinoma (HCC), promoting antigen presentation and stimulating antigen-specific CD8 + T-cell cytotoxicity [18]. Multiplex immunohistochemistry of human HCC samples and bioinformatic analysis of TCGA data further supported our findings that lower expression of *FASN* correlates with a higher percentage of cytotoxic CD8^+^ T cells [90]. The combination of two mechanistically distinct FASN inhibitors, namely orlistat and TVB-2640 [91–94], with an anti-PD-L1 antibody was found to robustly suppress tumor growth in vivo. Consistent with our current bioinformatic analysis of the immune cell landscape in the TCGA database, a recent comprehensive evaluation of the role of FASN in tumor immune infiltration and prognostic value for immunotherapy revealed a negative correlation of FASN with immune checkpoints (e.g., PD-1, PD-L1, CTLA-4) as well as a more favorable response to anti-PD-L1 treatment in FASN-overexpressing tumors [90]. All of these analyses strongly suggest that FASN may be explored not only as a novel predictive biomarker for immunotherapy, but also as a direct immunotherapeutic target.

Using CRISPR-based genetic loss-of-function approaches, we have shown that cancer cells are significantly more susceptible to cellular immune cytotoxicity when they are engineered to lack *FASN* gene and lipogenic function [37]. Because mathematical models of tumor-immune interactions can provide an analytical framework to address specific questions about tumor-immune growth and killing dynamics [95], we here took advantage of a macroevolutionary algorithm to simulate the extinction dynamics of cancer cell numbers upon interaction with CATs. The output data from the culture system was limited to cell number over time, and we therefore acknowledge that our mathematical deconvolution of the real-time killing assay data is only able to infer dynamics at this scale and dimension (i.e., cell number and time). Future studies with different experimental designs should examine how *FASN* loss affects more complex tumor-immune dynamics (e.g., nature of cell-based immunotherapy, heterogeneity of resistant and sensitive sub-populations, etc). However, it is noteworthy that our mathematical deconvolution revealed that *FASN* loss-induced sensitization to the cytotoxic activity of CATs paralleled a *FASN* loss-induced reduction in the carrying capacity of cancer cell populations. Our experimental conditions did not limit the availability of glucose and glutamine required to sustain proliferation of HAP1/HAP1 *FASN*-KO cells, and therefore the mechanistic basis for such a reduction in carrying capacity imposed by the absence of cancer cell-autonomous de novo FA synthesis cannot be masked by the supra-physiological nutrient levels characteristic of standard cell culture media. In this regard, we observed a strong functional relationship between the FASN status and the mitochondrial capacity to perform oxidative phosphorylation via the ETC, thus experimentally confirming one of the predicted cancer cell dependencies that mimics the consequence of depleting *FASN*. Our genetic and pharmacological approaches confirm and extend some of the previous evidence suggesting a close relationship between the functional status of FASN and the rate of oxidative phosphorylation in cancer and immune cells [96, 97]. In contrast to the *off-target* effects reported with many anticancer drugs that target genes required for cancer proliferation in a cell-autonomous manner [98], we previously found that the efficacy of the FASNi TVB-3166 was largely prevented by CRISPR/Cas9-driven *FASN* KO. Given the phenocopying effects of the CRISPR-driven *FASN* loss-of-function mutagenesis and the TVB-3166-driven pharmacological inhibition of FASN catalytic activity on mitochondrial OXPHOS dampening, our results strongly support a causal involvement of FASN signaling in the functional alterations of mitochondrial ETC *in cellulo*. However, we acknowledge that a direct effect of FASN on ETC function, rather than a mere association, would be clearly demonstrated in rescue experiments by reintroduction of FASN into *FASN* KO cells. Interestingly, the profoundly altered mitochondrial phenotype imposed by the genetic and pharmacological impairment of cytosolic FASN largely mimicked the ability of mitochondrial FAS (mFAS) to coordinate oxidative metabolism in mitochondria by regulating the assembly of several ETC complexes [99]. Since mFAS and cytosolic FASN build de novo FAs using the same chemistry [100], our findings certainly extend the emerging central role of endogenous FA biogenesis in directing mammalian oxidative metabolism. Given that acetyl-CoA is the substrate for mFAS and that (cytosolic) FASN can generate excessive amounts of free FAs that can be catabolized to acetyl-CoA [96, 97], it may be tempting to suggest that the FASN-driven abundance of FAs in cancer cells allows them to be an indispensable fuel source (in the sense that acetyl-CoA itself is catabolized) for mFAS-facilitated ETC assembly, cellular respiration, and downstream TCA function [98]. It should be further explored whether FASN acts as a key regulator of acetyl-CoA availability that, via mFAS, adjusts ETC complex levels accordingly to avoid the deleterious consequences of ETC function in the absence of substrate (e.g., excessive production of reactive oxidative species). Such an ability of FASN/mFAS to link anabolic FA synthesis with carbon fuel oxidation via regulation of ETC activity may be central to the reduced carrying capacity of the FASN-deficient cancer cell population and mediate the effects of FASN on cancer cell survival following cytolytic attack by T cells. Since cancer cells with elevated OXPHOS metabolism can form a barrier to T cell activity, promoting T cell exhaustion and decreasing antitumor immunity [101], further studies should explore whether FASN inhibitors, by acting as bona fide OXPHOS inhibitors targeting the mitochondrial ETC, may be particularly effective as immunotherapeutic agents in OXPHOS-dependent cancer subtypes.

Loss of *FASN* rendered cancer cells more susceptible to killing by an in vitro model of adoptive T-cell therapy (ACP), cytokine-induced non-MHC-restricted CAT cells [39–44], suggesting that antigen-specific tumor recognition by T cells is dispensable for the ability of FASN blockade to enhance the in vitro efficacy of ACP even at low (0.5:1), physiologically and therapeutically relevant effector-to-target ratios. How can the metabolic *tug-of-war* between tumor and cytolytic immune cells be unbalanced by suppression of FASN alone? An integrated systems approach aimed at identifying cancer genes that disfavor T-cell immunity revealed that up-regulation of anti-apoptotic proteins, particularly BCL-2 and BCL-X_L_, which have been widely implicated in drug resistance to chemotherapy and targeted therapies, also promotes tumor resistance to immunotherapy independent of PD-L1 expression levels [102]. Mechanistically, these anti-apoptotic proteins reduce the mitochondrial capacity to amplify the death signals delivered by T-cells by reducing the so-called mitochondrial priming –i.e., proximity to the mitochondrial apoptotic threshold– of cancer cells [20]. Accordingly, BH3 mimetics designed to block the sequestration capacity of anti-apoptotic BCL-2 proteins towards *BH3*-containing pro-apoptotic interactors sensitize cancer cells to NK- and T-cell-mediated killing [20]. We previously demonstrated that genetic or chemical ablation of FASN-driven lipogenesis elevates the primed state of cancer cells by upregulating pro-death BH3-only proteins such as BIM, PUMA, and NOXA, heightening mitochondrial priming and shifting cells toward a primed-for-death state [19]. Here, we speculated that cancer cells are pushed closer to apoptotic thresholds upon FASN loss (primed), thereby allowing less powerful hits or less frequent T-cell punches due to low E:T becoming sufficient to enable T-cell mediated destruction. Accordingly, we demonstrated that CATs and BCL-2/BCL-X_L_-targeting BH3 mimetics markedly synergized in killing *FASN* KO cancer cells, strongly supporting the notion that suppression of FASN metabolic signaling may make cancer cells easier targets for pro-apoptotic, cytotoxic signals from CATs by altering the BCL2 interactome. While alterations in cellular metabolism involving an elevation of OXPHOS have been shown to promote resistance to BH3 mimetics such as venetoclax, reduced mitochondrial respiration via impairment of ETC activity correlates with enhanced venetoclax sensitivity by increasing BCL2 dependency [50, 103, 104]. Mechanistically, loss of FASN resulting in ETC inhibition may similarly increase BCL-2/BCL-X_L_ dependency, thereby lowering the threshold for inducing apoptosis, making cancer cells easier to target for cytotoxic signals from CATs, and accelerating CAT-mediated killing.

Depending on the source of stimulation, the immune checkpoint PD-L1 can be categorized as inducible or constitutive. These correspond to adaptive and innate immune responses, respectively [66, 105, 106]. With regard to the former, a key mechanism by which cancer cells limit both the host immune response and the efficacy of adoptive cellular immunotherapies such as in vitro expanded CATs, cytokine-induced killer (CIK) cells, autologous tumor-infiltrating lymphocytes (TILs), and chimeric antigen receptor T and NK cells (CAR-T and CAR-NK) [107–112] is the reactive upregulation of PD-L1 in response to the IFNγ released by tumor-targeting T and NK cells [55, 56]. Regarding the latter, constitutive PD-L1 overexpression is driven and maintained by the oncogenic activation of endogenous pathways such as MAPK, PI3K/AKT/mTOR, and STAT1/3. In addition to monoclonal antibodies and small-molecules aimed at blocking the interaction of PD-L1 with PD-1 on the surface of cancer cells, the idea of “switching off” PD-L1 expression by preventing PD-L1 upregulation or PD-L1 translation inside the cell is attracting considerable research interest as a novel immunotherapeutic approach [104]. In particular, we are accumulating molecular and mechanistic perspectives for the development of new molecules that mediate the degradation of immature and mature PD-L1 during post-translational modification (PTM) stages such as PD-L1 palmitoylation [73, 80, 81]. Palmitoylation is a PTM required to maintain the stability of the PD-L1 protein by blocking its degradation [21, 22, 63, 70–73] and further enhances the integration of the cytoplasmic domain of PD-L1 into the cell plasma membrane [113]. Although the palmitoylated status of PD-L1 has been exclusively attributed to the catalytic activity of tissue cancer-specific palmitoyl acyltransferases containing Asp-His-His-Cys (DHHC) in the active center, it should be acknowledged that FASN activity may contribute to the de novo synthesis and intracellular accumulation of palmitoyl-CoA in the range of 0.1–10 μmol/L to serve as a palmitoylation donor of several cancer-related proteins (e.g., EGFR, MYD88, AKT, tubulin) [75–77, 114–117]. In our hands, CRISPR/Cas9-driven KO of *FASN* completely abolished PD-L1 palmitoylation while significantly preventing or reducing inducible and constitutive cell membrane-associated PD-L1 expression. This suggests that palmitoylated PD-L1 is a major component of the FASN-dependent acylated proteome through which FASN directly regulates PD-L1 protein stability and localization [113]. However, since palmitate depletion via FASN inhibition significantly alters cell membrane composition and function in tumor cells, it cannot be excluded that FASN suppression could promote dissociation of PD-L1 from the cell membrane to make its C272 palmitoylation site less accessible to DHHCs and substrate [112], thereby indirectly decreasing PD-L1 palmitoylation and increasing PD-L1 degradation.

The role of FASN as a novel tumor cell-intrinsic metabolic checkpoint that impairs T-cell immunity can be exploited to improve the clinical efficacy of immunotherapy. Strategies to improve and broaden the efficacy of ACTs are critical as they are curative for a limited number of cancer patients. To date, most if not all metabolic approaches proposed to improve the effectiveness of ACTs have aimed to prime more potent anti-tumor T cells, either by driving T-cell differentiation toward a more memory phenotype or by induction of a metabolic signature that enhances T-cell persistence and anti-tumor response in the nutrient-deprived TME [110]. In addition to exploiting the metabolic properties of ACTs themselves to overcome the short lifespan and persistence of adoptively transferred immune cells, pharmacological metabolic interventions aimed at targeting tumor-associated FASN may prime cancer cells for mitochondrial apoptosis, resulting in a more robust and durable response of FASN-dependent tumors to ACPs-mediated cytolysis. Controlling the intracellular life cycle of PD-L1 by targeting the post-translational palmitoylation step may provide a unique therapeutic avenue to improve the efficacy and personalize the use of ACPs and anti-PD-L1 therapy. In tumors where PD-L1 is reactively induced by IFNγ secreted by ACPs, FASN blockade, which disrupts the maturation of PD-L1 on the surface of cancer cells, can be used to overcome adaptive immune resistance. In tumors with innate immune resistance that constitutively express high levels of PD-L1, the benefits of monoclonal antibodies that competitively block PD-L1 on the cell surface may be diminished by the palmitoylation-dependent intracellular storage of PD-L1/PD-1 on recycling endosomes and their active redistribution to the cell membrane. Targeting PD-L1 palmitoylation via FASN suppression could potentially exhaust the intracellular storage of PD-L1/PD-L1, leading to its depletion and allowing for synergistic interactions in combination with available PD-L1-targeted therapies.

## Conclusions

Despite the encouraging clinical responses that have been achieved with immunotherapeutic approaches such as immune checkpoint inhibitors and ACT, the emergence of adaptive and innate immune resistance phenomena remains a critical barrier to extending the benefits of these therapies to more patients and cancer types. Our research describes FASN as a putative marker of immune evasion landscapes and suggests that this lipogenic enzyme may function as a constitutive mechanism that tumor cells use to resist and counteract cytolytic T-cell attack. By mechanistically linking two hallmarks of cancer, cellular metabolism and immune evasion, we add a lipogenic dimension to the growing body of evidence showing that certain tumor-intrinsic metabolic pathways may be sufficient to promote cancer immunoresistance. Targeting FASN may represent a novel strategy to circumvent cancer immune resistance that warrants clinical exploration to improve cancer susceptibility to T cell-based immunotherapies and immunotherapy in general.

## Materials and Methods

### Immune landscape analysis and cancer dependency map

The TCGA RNA-Seq and associated clinical data (RNA-Seq v2 with expectation maximization quantification, RSEM) were obtained from the Genomic Data Commons data portal (https://portal.gdc.cancer.gov). Gene expression values were log-transformed and the genes represented in less than 75% of the tumors in a given cancer type were filtered out. The CIBERSORTx algorithm [27, 28] was applied to the bulk gene expression profiles for the inference of the proportions of immune cell subsets. Gene expression signature levels or scores were calculated from the combined analysis of the corresponding gene components using the single-sample Gene Set Expression Analysis (ssGSEA) algorithm calculated in Gene Set Variation Analysis (GSVA; https://github.com/rcastelo/GSVA) [118, 119]. Multivariate regression analyses included TCGA study, age at diagnosis, tumor stage (I-II and III-IV) and subtype when appropriated, and were calculated in R software. The immune system gene signatures and features were compiled from the corresponding publications and uncertain gene names curated using EMSEMBL BioMart annotations [26, 30, 31, 120, 121].

The normalized CRISPR-knockout gene effects were obtained from the DedMap portal (version 12/22), and the multivariate regression analysis included the covariates of cancer type (OncoTree), *PI3KCA* and *TP53* mutation status (0/1), and expression (normalized RNA-seq data) of *CCND1*, *MKI67*, and *MYC*. The pre-ranked (β gene values) GSEA algorithm was used with the default parameters against the signature gene set.

To assess the relationships between differential expression of *FASN* with immune cell content and immune escape signatures, the first tertile (percentile 0.33) and second tertile (percentile 0.66) of *FASN* expression were calculated for the TCGA BRCA tumors. On the basis of these values, the tumors were clustered into three groups, namely low, medium, and high *FASN* expression groups. For these three groups, hypothesis testing was performed with respect to each parameter of interest. The null hypothesis was the equality of the means between the groups, with the alternative being the non-equality of all the means.

### Cell lines and culture conditions

HAP1 and HAP1 *FASN-KO* cells (#HZGHC003700c006) were purchased from Horizon Discovery Ltd. (Cambridge, UK) and routinely grown in IMDM medium (Gibco) supplemented with 10% FBS, 2 mmol/L L-glutamine, and 100 IU/mL penicillin/streptomycin.

JIMT-1 cells were obtained from the German Collection of Microorganisms and Cell Culture (Braunschweig, Germany) and grown in Dulbecco’s modified Eagle’s medium (DMEM) supplemented with 10% heat-inactivated fetal bovine serum (FBS; BioWhittaker Inc., Walkersville, MD), 1% L-glutamine, 1% sodium pyruvate, 50 U/mL penicillin, and 50 μg/mL streptomycin.

CRISPR-based JIMT-1/*FASN* KO cells were generated using an Edit-R pre-designed all-in-one lentiviral sgRNA. Briefly, lentiviral particles were generated using HEK293T cells at 60–70% confluence transfected with Addgene lentiviral plasmids (plasmids pCMV-dR8.2 and pCMV-VSV-G, Cat.#8455 and #8454, respectively), Horizon’s Edit-R Human FASN hEF1a All-in-one lentiviral sgRNA (Cat.#GSGH11935-247546240, Horizon Discovery) in the presence of Fugene 6 (Cat.#E2691; Promega). The next day, the medium was changed, and 24 h later, conditioned media containing lentiviral particles were collected and filtered. Lentiviral particles were then used to transduce JIMT-1 cells with 8 μg/mL polybrene (Cat.#TR-1003-G, Merck) and cells were selected with 10 μg/mL puromycin (Cat.#P8833, Sigma) 48 h after viral infection.

All cells were maintained at 37^o^C in a humidified atmosphere of 95% air and 5% CO_2_. Cells were screened periodically for the absence of *Mycoplasma* using the MycoAlert^®^ Mycoplasma Detection Kit (Lonza, Walkersville, MD).

### Immunoblotting

Cells were rinsed once in ice-cold PBS and harvested in a lysis buffer containing 150 mmol/L NaCl, 50 mmol/L Tris-HCl pH 7.4, 1 mmol/L EDTA, 1% Triton-X 100, 1 mmol/L phenylmethylsulfonyl fluoride, 1 mmol/L Na_3_VO_4_. Samples were sonicated for 1 min (under ice water bath conditions) with 2 s of sonication at 2 s intervals to completely lyse cells and reduce viscosity. The Bradford protein assay (Bio-Rad, Hercules, CA, USA) was used to determine protein content. Equal amounts of cellular protein were electrophoresed on 12% SDS-PAGE gels, transferred to nitrocellulose membranes, and probed with primary antibodies to FASN (Cat. #610963, BD Transduction Laboratories™/BD Biosciences, San Jose, CA) or PD-L1 (E1L3N^®^ XP^®^ rabbit mAb #13684, Cell Signaling Technology, Danvers, MA), followed by incubation with a horseradish peroxidase-conjugated secondary antibody, and chemiluminescence detection. β-actin (Cat. #66009-1-Ig, Clone #2D4H5, Proteintech Group, Inc., Rosemont, IL) was used as a control for protein loading.

### Cytokine-activated T cells

To obtain cytokine-activated T cells (CATs), human peripheral blood mononuclear cells (Cat. #70025) were cultured in ImmunoCult-XF™ T cell expansion medium (Cat. #10981) containing ImmunoCult-XF™ Human CD3/CD28/CD2 T cell activator (Cat. #10971) (all from StemCell Technologies, Vancouver, BC, Canada), and 10 ng/mL IL-2 (Cat. #200-02, PeproTech, Rocky Hill, NJ) for 1 week according to the manufacturer’s instructions. Cell-based immunotherapy assays with CATs were performed in the presence of anti-CD3 antibody (100 ng/mL; Cat. #16-0037; eBioscience, Thermo Scientific) and IL-2 (10 ng/mL).

### Cell-based immunotherapy potency assays

The impact of *FASN* loss on the efficacy of cell-based immunotherapy was evaluated using the xCELLigence Real-Time Cell Analysis (RTCA) system. The output of xCELLigence is the so-called Cell Index (CI) parameter, which is calculated from changes in electrical impedance in the culture plate over time and is highly correlated with the number of cells in the well/plate, as well as with changes in cell size and cell-substrate attachment strength. Impedance (or resistance) to an electric current occurs when adherent target (T) cancer cells bind to the surface of the gold electrodes in each well of an Agilent “E-Plate 16”, thereby impeding the flow of an electric current between the electrodes; conversely, the electrical impedance signal is reduced when cancer cells detach after being killed by cytolytic immune cells. In contrast to surface-attached cancer cells, immune effector (E) cells (e.g., CATs) do not directly affect the impedance signal because they are not in contact with the electronic sensors. However, killing of tumor cells results in tumor cell detachment or disintegration events that can be sensitively and accurately detected in terms of reduced electrical impedance. Because of these different properties, the cytolytic activity of CATs can be selectively monitored in real time using the RTCA system (Fig. S1).

Briefly, the impedance of CRISPR/Cas9-based *FASN* + */FASN KO* isogenic cancer cells added to E-Plates 16 and placed in the xCELLigence^®^ RTCA DP station was measured every 5 min for 20–24 h until the cells adhered to the gold electrodes at the bottom of each well. Then, CATs at various ratios (e.g., 0.5:1, 1:1, 2:1, 5:1) or media alone were then added to the respective wells and the xCELLigence RTCA assay was then restarted to continuously acquire impedance measurements every 5 min for an additional 48–72 h using the RTCA Software 1.2 (ACEA Biosciences). After normalization of the CI data to account for “target” cancer cells alone and “effector” CATs alone, parameters such as % cytolysis (=[CI_no effector_-CI_effector_]/CI_no effector_ × 100]) and the so-called “Killing Time” (KT), which represents the time required to achieve a given % cytolysis at a given E:T ratio, were determined using the xIMT software (ACEA Biosciences).

The proliferation growth regimes from cell-based immunotherapy potency assays were used to fit the mathematical model (see below) and quantify the dynamics of CATs cell killing of *FASN+* cancer cells compared to *FASN KO* counterparts.

### Mathematical model

The dynamics of cancer-T cells under the experimental conditions described above were mathematically modeled using differential equations describing the rate of variation of each cell type population over time according to the law of mass action [122, 123]. The variables are given by a heterogeneous population of *M* cancer cells (variable {$y$_i_}*, i* = *1, …, M*) and T cells (variable $x$), which are divided into active T cells (variable $x$_A_) and exhausted T cells (variable $x$_E_). Active T cells can induce cancer cytolysis and are exhausted by this process, becoming nonfunctional T cells. The model assumes that different cancer cell populations can be found in the experimental setup due to intrinsic variability (e.g., size, attachment/spreading ability, proliferation kinetics, etc). A second assumption is that the cancer cell populations grow according to a logistic model, where cells multiply exponentially at low population numbers and compete for both the physical space and the resources available on the plate as they increase in population. Active T cells are introduced at the end of the exponential growth phase of cancer cells, and they lose their cytolytic potency until they are exhausted. The model also assumes that T cells, both active and exhausted, do not compete with cancer cells for physical space during proliferation or loss of effector cytolytic function in suspension.

The dynamics of the cancer cell populations, $y$_i_, is given by their proliferation, which is limited by the competition among cancer cells, together with the cell death and cytolysis caused by active T cells, and can be read as the following dynamic equation: $\dot{y}$_*i*_ *= proliferation* $y$_i_ *– degradation* $y$_i_ *– cytolysis of* $y$_i_ *induced by* $x$_A_*, i* = *1,…,M*, where the dot above the variable denotes the time derivative. With respect to T cells (active, $x$_A_; and exhausted, $x$_E_), whose residual populations were not measured by any means throughout the experimental procedure, the model includes both proliferation and exhaustion of active CATs and removal of exhausted T cells, according to the following differential equations: $\dot{x}$_A_ = proliferation *A* – exhaustion, $\dot{x}$_E_ = exhaustion – degradation E. Table 1 lists all of the parameters of the mathematical model, including the biological significance, their biological meaning, the ranges explored, and the values obtained from the best fits to the experimental data.

**Table 1:** Mathematical model parameters.

Parameter	Significance	Range
*r*	Proliferation rate of cancer cells	0–50
*D*	Degradation rate of cancer cells	0–50
*a*	Logistic weight of cancer cells	10^-3^–1
*l*	Exhaustion rate of T cells	0–5
*h*	Cytolytic effect rate of T cells	0–5
*b*	Logistic weight of T cells	10^-3^–1
*r*_*A*_	Proliferation rate of active T cells	0–50
*d*_*E*_	Degradation rate of exhausted T cells	0–50
*K*	Carrying capacity of cancer cells	1–100
*C*	Carrying capacity of T cells	1–100

Units for the rates are *h*^−^^1^, competition coefficients are dimensionless, and carrying capacities are expressed in terms of cell index (CI) values as a proxy for cell population size.

Therefore, the model for each population can be written in the following way:

$$\begin{array}{ll}{Cancer}\; {cell}\; {dynamics}&{\dot{y}}_{{\rm{i}}}={Y}_{i}\left(x\right)={r}_{i}{y}_{i}\left(1-\frac{{\sum }_{j=1}^{M}{a}_{j}{y}_{j}}{K}\right)\\&\qquad\;-\,{d}_{i}{y}_{i}-{h}_{i}{y}_{i}{x}_{A},\,i=1,\ldots ,M\end{array}$$

$${Active}\,{T}\,{cells}\,\,{\dot{x}}_{{\rm{A}}}={X}_{A}\left(x\right)={r}_{A}{x}_{A}\left(1-\frac{{\sum }_{j{\rm{\in }}\left\{A{\rm{;}}E\right\}}{b}_{j}{x}_{j}}{C}\right)-{x}_{A}\mathop{\sum }\limits_{j=1}^{M}{l}_{j}{y}_{j}$$

$${Exhausted}\,{T}\,{cells}\,{\dot{x}}_{{\rm{E}}}={X}_{E}\left(x\right)={x}_{A}\mathop{\sum }\limits_{j=1}^{M}{l}_{j}{y}_{j}-{d}_{E}{x}_{E}$$

Different values of *M* were first tested to find the minimum number of cancer cell populations that would allow a good fit. Based on three well-defined growth phases in the experimental data, the number of cancer cell populations was set to *M* = *3*, which gives a good fit with a very small error.

### Numerical tools

A fourth-order Runge-Kutta method with adaptive time step size was used to numerically solve the differential equations.

### Experimental data fitting and parameters estimation

The mathematical model described above was fitted to the experimental data sets. The parameter ranges (Table 1) were kept large enough to allow a large volume search of the parameter space. Macroevolutionary algorithms (MA) were used to fit the model to the data. These algorithms provide a heuristic optimization method that performs well on rough fitness landscapes [45, 46]. These algorithms consider a population of parameter vectors, and each of these vectors is assigned a fitness value by computing a distance measure between experimental and imulated data with the mathematical model. Here, the distance is computed using the least squares method (LS). The goal of MA is therefore to optimize this distance by minimizing it.

The MA works as follows. A population Ω (N,τ) of N vectors of parameters is defined (we used *N* = 1000), where τ are the generations of the MA. For τ = 0, Ω (N) is initialized by randomly choosing the values of the parameters within the ranges given in Table 1 using uniform distributions. Then the MA iteratively follows the next steps:

1. The equations are solved numerically using the parameter values of each vector of the population Ω (N), and the LS between the experimental and the simulated data is computed for each vector.
2. The vectors are ordered from lowest to highest LS values, i.e., from higher to lower fitness, and the 25% of the vectors with the higher fitness of Ω (N,τ), denoted as ***p***, are selected as part of the initial population of vectors of the population at the next generation Ω (N,τ + 1).
3. To complete Ω (N, τ + 1), an additional 25% of the population of vectors ***p’*** is included by slightly modifying the population ***p*** with small random perturbations obtained from ***p’*** = ***p*** *(1* + *β)*, where *β* is a random number obtained from a uniform distribution with $\beta \in U(-\mathrm{0.02,0.02}).$ The remaining 50% are again randomly selected within the ranges defined in Table 1.
4. Return to 1.

This algorithm allows for a wide search for good solutions in the parameter space and a fine tuning of such solutions. Thus, this process continuously selects and improves the good vectors that provide the lowest LS values and thus greater fitness, along with macro-extinctions of those vector populations that do not provide good solutions. In this way, the algorithm performs well in finding the global minimum in the parameter space, i.e., LS → 0. The algorithm stops when the boundary number of iterations is reached (we set the maximum number of MA generations to τ = 10^4^ to fit the cancer growth parameters, τ = 1000 to fit for all the other parameters). The intrinsic parameters for cancer cell dynamics (*r*, *d*, *α*) were fitted using experimental data for cancer cell populations in the absence of T cells, i.e., a dynamic model without the *x*_*A*_ and *x*_*E*_ equations. The fits used the best 250 parameter vectors obtained in each run of the MA. Using the optimized parameters as structural parameters of the cancer cell populations, we fitted the filling model with experimental data in the presence of T cells using the same procedure described above.

### Extracellular flux assay

The effects of *FASN* gene knockout on mitochondrial function were determined using the XF24 Seahorse Biosciences Extracellular Flux Analyzer (Agilent Seahorse Technologies). Cells growing in regular media were plated at a density of 7500 cells/well on XFp cell culture miniplates (Seahorse XFp FluxPak, Cat. #103022-100, Agilent Seahorse Technologies) and grown overnight at 37 ^o^C with 5% CO_2_ in a humidified incubator. The media was then removed and the cells were washed and incubated with pre-warmed assay media (XF Base Medium Minimal DMEM containing 10 mmol/L glucose, 1 mmol/L sodium pyruvate, and 2 mmol/L glutamine) for 1 h in a non-CO_2_ incubator at 37^o^C. The Seahorse XFp Cell Mito Stress Test Kit (Cat. #103010-100, Agilent Seahorse Technologies) was used to determine OCR with sequential treatment with 1.5 μmol/L oligomycin A, 1 μmol/L FCCP, and 0.5 μmol/L rotenone/antimycin A. OCR data were normalized to cell number.

### Crystal violet cell killing assay

Cells were seeded in 12-well plates at a density of 60,000 cells/well. After an overnight attachment period, cells were treated with ABT-263/navitoclax (Cat. #S1001, Selleckchem, Houston, TX), ABT-199/venetoclax (Cat. #S8048, Selleckchem, Houston, TX), A1331852 (Cat. #S7801, Selleckchem, Houston, TX), S63845 (Cat. #S8383, Selleckchem, Houston, TX) and/or CATs for 48 h. Vehicle- and drug/CAT-treated cells were washed twice with cold PBS and incubated with 0.5% (*w/v*) crystal violet solution in 25% methanol for 20 min. The methanol was aspirated and the cells were incubated with 0.5% crystal violet solution in 25% methanol for 20 min. The cells were washed several times and dried overnight.

### PD-L1 immunofluorescence

Cells were seeded on glass coverslips and then fixed with 4% paraformaldehyde in phosphate-buffered saline (PBS). After fixation for 5 min at room temperature (RT), the cells were permeabilized with 0.1% Triton X100/PBS. Coverslips were then placed in the antibody solution (PD-L1 extracellular domain-specific E1J2J rabbit mAb #15165 1:100 dilution; Cell Signaling Technology, Danvers, MA) and incubated at RT for 60 min. Cells were washed and stained with a secondary antibody. Nuclei were counterstained with Hoechst 33342. Images were captured using an Eclipse 50i fluorescence microscope equipped with NIS-Elements imaging software (Nikon, Tokyo, Japan).

### PD-L1 palmitoylation

Evaluation of FASN-regulated PD-L1 palmitoylation was performed using the commercially available CAPTUREome™ S-palmitoylated protein kit (Cat. #K010-311, Badrilla, UK) according to the manufacturer’s instructions. The assay is based on the acyl resin-assisted capture (RAC) methods and facilitates the determination of post-translational protein modification with a palmitate group using four steps, namely free thiol blockade, thioester bond cleavage, nascent thiol capture on Sepharose, and analysis [78]. Briefly, equal amounts of protein (1–2 mg) were added to 500 μL of blocking buffer (buffer A with thiol blocking reagent) and shaken at 40 ^o^C for 4 h. Proteins were precipitated at −20 ^o^C for 20 min with the addition of three volumes of cold acetone. After centrifugation of the solution at 16,000 g for 5 min, the pellet was thoroughly washed five times with 70% acetone and completely air dried after the last wash. The pellet was redissolved in 300 μL binding buffer and incubated for 1 h at 40 ^o^C in a shaking heat block. The homogenates were centrifuged at 16,000 g for 5 min to remove insoluble debris, and approximately 20 μL of each supernatant was saved as the “total input”. The pre-washed capture resin slurry (50 μL) was added to the remaining lysates, followed by the addition of 19 μL of thioester cleavage reagent. The binding reactions were performed on a rotator at room temperature for 2.5 h. The resins were washed a minimum of five times with the binding buffer. The supernatants were removed and mixed with 2×Laemmli loading buffer, heated to 60 ^o^C for 10 min, and resolved by SDS-PAGE.

### Statistical analysis

At least three independent experiments with *n* ≥ 3 replicate samples per experiment were performed for all experiments. Data are presented as mean ± S.D. Bar graphs, curves, and statistical analyses were generated using the R package 4.4.1 and the GraphPad PRISM 10 (GraphPad Software, Inc., San Diego, CA). Two-group comparisons were performed using Student’s *t*-test for paired and unpaired values. Comparisons of the means of ≥ 3 groups were performed by ANOVA, and the existence of individual differences, in the case of significant values in ANOVA, was tested by multiple contrasts. Statistical tests were two-tailed.

## Supplementary information

Supplementary information

Uncropped original immunoblottings

## Data Availability

All of the data sets used in the present study are available from the corresponding authors upon reasonable request.
