# Supplementary material for: Fatty acid synthase (FASN) is a tumor-cell-intrinsic metabolic checkpoint restricting T-cell immunity
Source: Cell Death Discov. 2024 Sep 30;10:417. doi: 10.1038/s41420-024-02184-z (PMC11442875; doi:10.1038/s41420-024-02184-z)
Supplement: Supplementary file 1 — Supplementary information [file 41420_2024_2184_MOESM1_ESM.docx]

**SUPPLEMENTARY INFORMATION**

**Table S1. *FASN* expression and immune cell contents: correlation coefficients and statistical significance**

| **Term** | **Spearman** | **Spearman.pval** | **Spearman.padj** | **Pearson** | **Pearson.pval** | **Pearson.padj** |
| --- | --- | --- | --- | --- | --- | --- |
| B cells memory | -0,14080838 | 3,0283E-06 | 1,5593E-05 | -0,13856746 | 4,3513E-06 | 2,149E-05 |
| B cells naive | 0,02047714 | 0,49925567 | 0,56990505 | -0,00808122 | 0,78975922 | 0,87270197 |
| Dendritic cells activated | -0,11735308 | 0,00010225 | 0,00034367 | -0,08532477 | 0,00479914 | 0,0113862 |
| Dendritic cells resting | 0,02194759 | 0,46894816 | 0,54040692 | 0,00257769 | 0,93222619 | 0,96403652 |
| Eosinophils | -0,03955996 | 0,19165792 | 0,27123518 | -0,06370469 | 0,03538635 | 0,06390669 |
| Macrophages M0 | -0,00691226 | 0,81960352 | 0,84118489 | -0,01977596 | 0,51406526 | 0,61892434 |
| Macrophages M1 | -0,08670619 | 0,00415633 | 0,00957935 | -0,09762546 | 0,00124391 | 0,0035003 |
| Macrophages M2 | 0,12044367 | 6,6561E-05 | 0,00023246 | 0,09682718 | 0,00136434 | 0,00379507 |
| Mast cells activated | -0,03920641 | 0,19566122 | 0,27529078 | -0,03330526 | 0,27171305 | 0,37574033 |
| Mast cells resting | 0,24253798 | 4,529E-16 | 1,096E-14 | 0,18994215 | 2,5437E-10 | 2,9313E-09 |
| Monocytes | 0,01521067 | 0,61576195 | 0,67733814 | -0,02406995 | 0,42705519 | 0,53710664 |
| Neutrophils | 0,06060684 | 0,04534859 | 0,07449765 | 0,00864177 | 0,77555359 | 0,86490308 |
| NK cells activated | -0,02729115 | 0,36781749 | 0,45882388 | -0,02425802 | 0,42345088 | 0,53651892 |
| NK cells resting | 0,02558714 | 0,39849065 | 0,48335817 | -0,01939407 | 0,52222625 | 0,62073606 |
| Plasma cells | 0,08211189 | 0,00665456 | 0,01477435 | 0,05863024 | 0,05286534 | 0,09138151 |
| T cells CD4 memory activated | -0,10321692 | 0,00063899 | 0,00179809 | -0,12006826 | 7,0163E-05 | 0,00027386 |
| T cells CD4 memory resting | 0,01832901 | 0,54533237 | 0,60493809 | 0,01376599 | 0,64968854 | 0,74514041 |
| T cells CD4 naive | -0,06299235 | 0,03749466 | 0,06345251 | -0,04066789 | 0,17950279 | 0,26011782 |
| T cells CD8 | -0,05464284 | 0,07120814 | 0,11046391 | -0,07111902 | 0,0188055 | 0,03699944 |
| T cells follicular helper | -0,07964585 | 0,0084911 | 0,01786822 | -0,08464852 | 0,00514547 | 0,01185908 |
| T cells gamma delta | -0,12743898 | 2,4242E-05 | 9,6172E-05 | -0,1386846 | 4,2702E-06 | 2,149E-05 |
| T cells regulatory (Tregs) | 0,02397221 | 0,42893514 | 0,50676535 | 0,01730065 | 0,5681128 | 0,66097739 |
|  |  |  |  |  |  |  |
| CYT | -0,12520538 | 3,6889E-05 | 0,00016005 | -0,1332936 | 1,1076E-05 | 4,9231E-05 |
| HLAI | -0,13799014 | 5,3301E-06 | 2,7124E-05 | -0,10792444 | 0,00038082 | 0,00125853 |
| HLAII | -0,08491448 | 0,00523206 | 0,01359837 | -0,05887731 | 0,05307093 | 0,10033723 |
|  |  |  |  |  |  |  |
| Ayers_18TIS_excl_ConsTME | -0,12464645 | 3,5329E-05 | 0,00012295 | -0,12793841 | 2,1742E-05 | 7,464E-05 |
| Ayers-IFNg_sign | -0,14633695 | 1,1558E-06 | 5,06E-06 | -0,14082971 | 2,8944E-06 | 1,1271E-05 |
| Ayers-infl-18TIS-sign | -0,14957524 | 6,6299E-07 | 2,9985E-06 | -0,15056393 | 5,582E-07 | 2,4298E-06 |
| Frøssing_infl-asthma | -0,02099547 | 0,48765743 | 0,58213495 | -0,02970143 | 0,32613047 | 0,41806591 |
| GOBP_CHRONIC_INFLAMMATORY_RESPONSE | -0,15162476 | 4,6354E-07 | 2,1705E-06 | -0,15286941 | 3,7212E-07 | 1,6526E-06 |
| GOBP_IMMUNE_RESPONSE | -0,17398685 | 6,8305E-09 | 4,088E-08 | -0,17509926 | 5,4547E-09 | 3,0938E-08 |
| GOBP_INFLAMMATORY_RESPONSE | -0,15183519 | 4,4669E-07 | 2,0999E-06 | -0,14749537 | 9,4867E-07 | 3,9841E-06 |
| GOBP_RESPONSE_TO_TYPE_I_INTERFERON | -0,07453045 | 0,01362938 | 0,02796974 | -0,0561842 | 0,06309466 | 0,10489392 |
| Hu_NatComm_TH1-17_infl-sign | -0,1612534 | 8,0925E-08 | 4,1887E-07 | -0,17237301 | 9,4418E-09 | 5,1624E-08 |
| Hu_NatComm_TH1-17_infl-sign_excl_ConsTME | -0,1541738 | 2,9504E-07 | 1,4192E-06 | -0,16118949 | 8,1896E-08 | 3,9776E-07 |
| Jiang_CAF | -0,11044938 | 0,00025036 | 0,0007407 | -0,08833243 | 0,00344046 | 0,00786539 |
| Jiang_ICB_resist | -0,22339952 | 7,5041E-14 | 7,5393E-13 | -0,20893599 | 2,8859E-12 | 2,4191E-11 |
| Jiang_MDSC | -0,25184147 | 2,6544E-17 | 3,1659E-16 | -0,23867579 | 1,1955E-15 | 1,3729E-14 |
| Jiang_T_accum | -0,24731355 | 1,0085E-16 | 1,1776E-15 | -0,24283325 | 3,6805E-16 | 4,4342E-15 |
| Jiang_T_exhaust | -0,14192915 | 2,4163E-06 | 1,0107E-05 | -0,13428335 | 8,2468E-06 | 3,0252E-05 |
| Jiang_T_regulatory | -0,12783171 | 2,2091E-05 | 7,943E-05 | -0,12811694 | 2,117E-05 | 7,2825E-05 |
| Jiang_TAM_M2_M1 | -0,15199749 | 4,341E-07 | 2,0453E-06 | -0,14494924 | 1,4612E-06 | 5,9716E-06 |
| Prueitt-infl-immu | -0,0966475 | 0,00136483 | 0,00347302 | -0,04489364 | 0,13764514 | 0,20386717 |
| Prueitt-infl-prostsmok | -0,17365577 | 7,3014E-09 | 4,3388E-08 | -0,18013186 | 1,9363E-09 | 1,1588E-08 |
| Prueitt-infl-prostsmok_excl_ConsTME | -0,18927948 | 2,7296E-10 | 1,9428E-09 | -0,19296041 | 1,2064E-10 | 8,4149E-10 |
| Thorsson_InhibitoryCheckpoint | -0,19718327 | 4,6346E-11 | 3,5964E-10 | -0,18556065 | 6,1264E-10 | 3,9105E-09 |
| Thorsson_StimulatoryCheckpoint | -0,18742029 | 4,0976E-10 | 2,825E-09 | -0,18614868 | 5,3972E-10 | 3,4742E-09 |
| WP_INFLAMMATORY_RESPONSE_PATHWAY | -0,14868447 | 7,7341E-07 | 3,4685E-06 | -0,15282178 | 3,7527E-07 | 1,6657E-06 |

**Table S2. Multivariate regression analysis of**

***FASN* and TCGA immune signatures**

| **Term** | **Estimate** | **Std error** | ***t–*value** | ***p–*val** |
| --- | --- | --- | --- | --- |
| cancerTypeTHCA | -1.7798873 | 0.05553326 | -32.050837 | 5.04E-205 |
| cancerTypeKIRC | -1.6630894 | 0.06984906 | -23.80976 | 9.68E-119 |
| (Intercept) | 4.88688319 | 0.31032793 | 15.7474812 | 1.47E-54 |
| cancerTypePAAD | -1.0064697 | 0.06817616 | -14.76278 | 2.66E-48 |
| cancerTypeLIHC | 0.80151882 | 0.05898742 | 13.5879629 | 2.57E-41 |
| cancerTypeBRCA | 0.65359797 | 0.04876858 | 13.4020312 | 2.93E-40 |
| T.cells.gamma.delta | -5.3425555 | 0.61257055 | -8.721535 | 3.69E-18 |
| B.cells.memory | -5.1920644 | 0.74683534 | -6.9520872 | 4.07E-12 |
| T.cells.CD4.memory.activated | -2.604931 | 0.39411425 | -6.6095834 | 4.26E-11 |
| Dendritic.cells.resting | -2.7613065 | 0.50136292 | -5.5076002 | 3.82E-08 |
| cancerTypeSKCM | 0.46606708 | 0.0900927 | 5.17319488 | 2.39E-07 |
| cancerTypeESCA | 0.33602621 | 0.06953394 | 4.83254923 | 1.39E-06 |
| T.cells.CD4.memory.resting | -1.3882034 | 0.33239889 | -4.1763177 | 3.01E-05 |
| cancerTypeHNSC | -0.2216195 | 0.05315213 | -4.1695317 | 3.10E-05 |
| NK.cells.activated | -2.3082625 | 0.61406172 | -3.7590074 | 0.00017255 |
| Macrophages.M2 | -1.2371346 | 0.35515849 | -3.483331 | 0.00049949 |
| Monocytes | -1.7713698 | 0.55376886 | -3.198753 | 0.00138892 |
| Macrophages.M1 | -1.4636029 | 0.4626319 | -3.1636446 | 0.00156748 |
| Macrophages.M0 | -0.961874 | 0.34877646 | -2.7578523 | 0.00583955 |
| T.cells.CD8 | -1.0965601 | 0.40352712 | -2,7174383 | 0.00660179 |
| stageLHLow | -0.0514811 | 0.02178971 | -2.3626333 | 0.018184 |
| cancerTypeLUSC | -0.1213951 | 0.05515535 | -2.2009663 | 0.02778431 |
| Mast.cells.activated | -1.0273438 | 0.47573721 | -2.1594775 | 0.03086091 |
| Plasma.cells | -0.7832448 | 0.38775728 | -2.0199357 | 0.04344371 |
| cancerTypeCOAD | -0.109996 | 0.05586784 | -1.9688606 | 0.04902468 |
| Neutrophils | -1.2864238 | 0.7755778 | -1.6586651 | 0.09724651 |
| NK.cells.resting | 0.84176946 | 0.52111116 | 1.61533571 | 0.1063016 |
| B.cells.naive | -0.6885827 | 0.43776638 | -1.5729457 | 0.11579517 |
| cancerTypeLUAD | -0.0972253 | 0.06326897 | -1.5366974 | 0.1244312 |
| Dendritic.cells.activated | -0.7704405 | 0.54665836 | -1.4093638 | 0.15879031 |
| T.cells.follicular.helper | 0.71947878 | 0.56848849 | 1.26559955 | 0.20571592 |
| cancerTypeSTAD | 0.05988367 | 0.05405108 | 1.10790891 | 0.26795487 |
| age | -0.0006531 | 0.00079353 | -0.8230611 | 0.41051281 |
| Mast.cells.resting | -0.2987362 | 0.38249803 | -0.7810136 | 0.43483177 |
| cancerTypeTGCT | -0.0552237 | 0.08273572 | -0.6674713 | 0.50450219 |
| Eosinophils | -0.5911632 | 0.89733611 | -0.658798 | 0.51005609 |
| T.cells.CD4.naive | 0.42652042 | 1.20177677 | 0.35490819 | 0.72267346 |

**
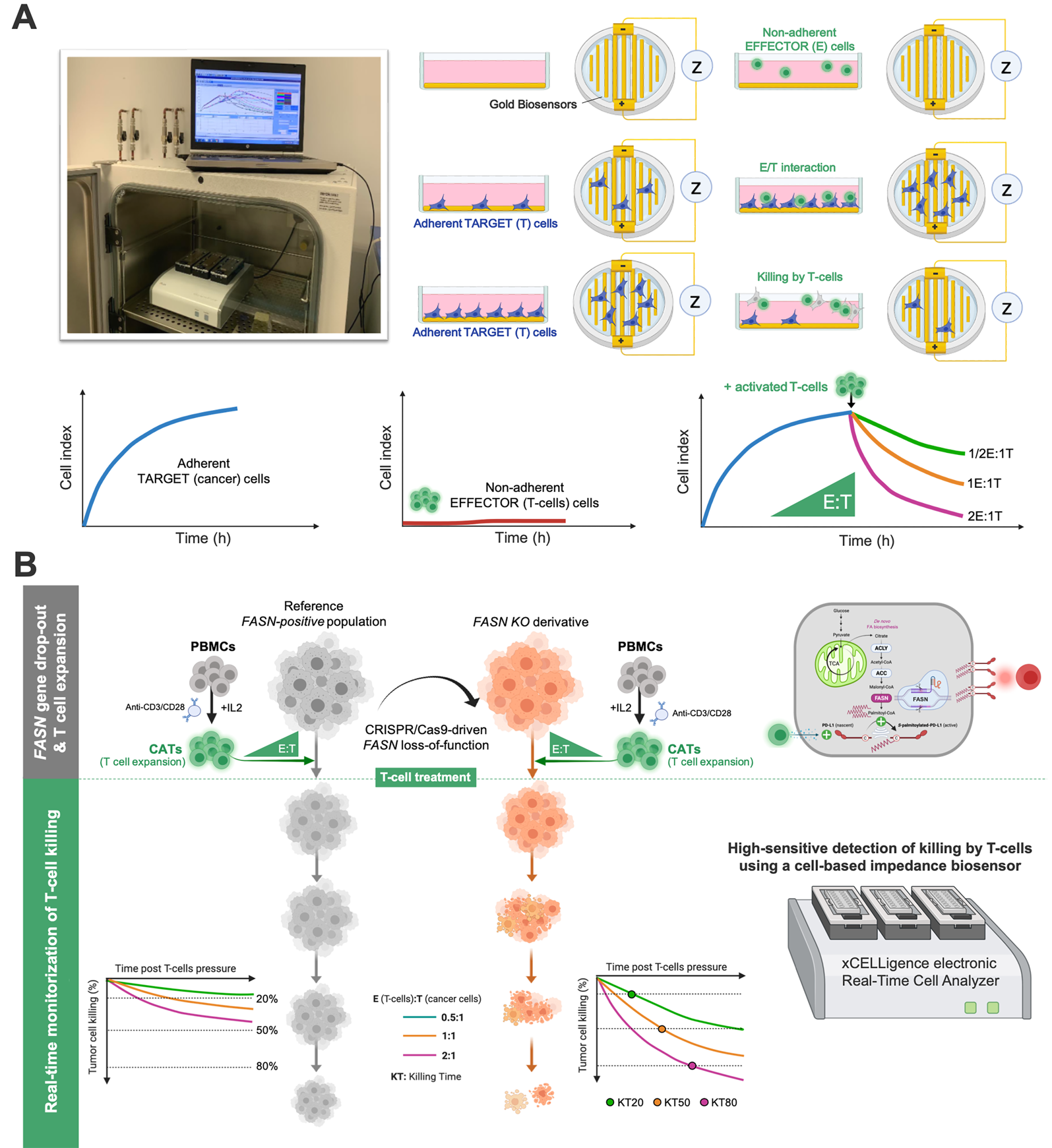
**

**Figure S1. Deconvolution of FASN-driven immune resistance using real-time killing assay data.** Schematic of experimental design and output data from combining real-time monitoring of T-cell killing using the xCELLigence system with CRISPR-Cas9 technology for functional *FASN* gene immunoscreening. **A.** Functional principle of the xCELLigence impedance technology applied to cell-based immunotherapy monitoring. The xCELLigence RTCA label-free technology monitors cell counts through changes in impedance (*Z*) measured by gold electrodes embedded in proprietary E-Plates. When seeded alone, the proliferation rate of adherent target (T) cancer cells is recorded as an increase in the so-called impedance-related Cell Index (CI) parameter over time (free electron flow, CI=0; impeded electron flow, CI>0), reaching a plateau as cells approach 100% confluence. Effector (E) non-adherent immune cells produce a low baseline level signal due to the lack of tight surface adhesion over the gold electrodes. When E immune cells (e.g., cytokine-activated T cells in this work) are added to adherent T cells, their cytolytic activity causes the adherent cells to round up and detach, resulting in a reduction in the CI values. Real-time killing curves can be generated for multiple conditions simultaneously by continuous acquisition of impedance data. **B.** To functionally interrogate the contribution of tumor-intrinsic metabolic pathways such as FASN-driven *de novo* FA biosynthesis to tumor immune evasion, we took advantage of CRISPR-Cas9 technology for functional immunoscreening of the *FASN* gene. Here, we show schematically of how CRISPR/Cas9-based knockout (KO) of *FASN*, combined with automated, real-time measurements of the cytolytic interactions between T cells and FASN-positive/FASN-KO cancer cells at different effector-to-target ratios (as representative levels of T cell selection pressure), can unbiasedly identify cancer cell-autonomous lipogenic traits as tumor-intrinsic factors that regulate sensitivity to T cell-mediated killing in cancer immunotherapy.

**
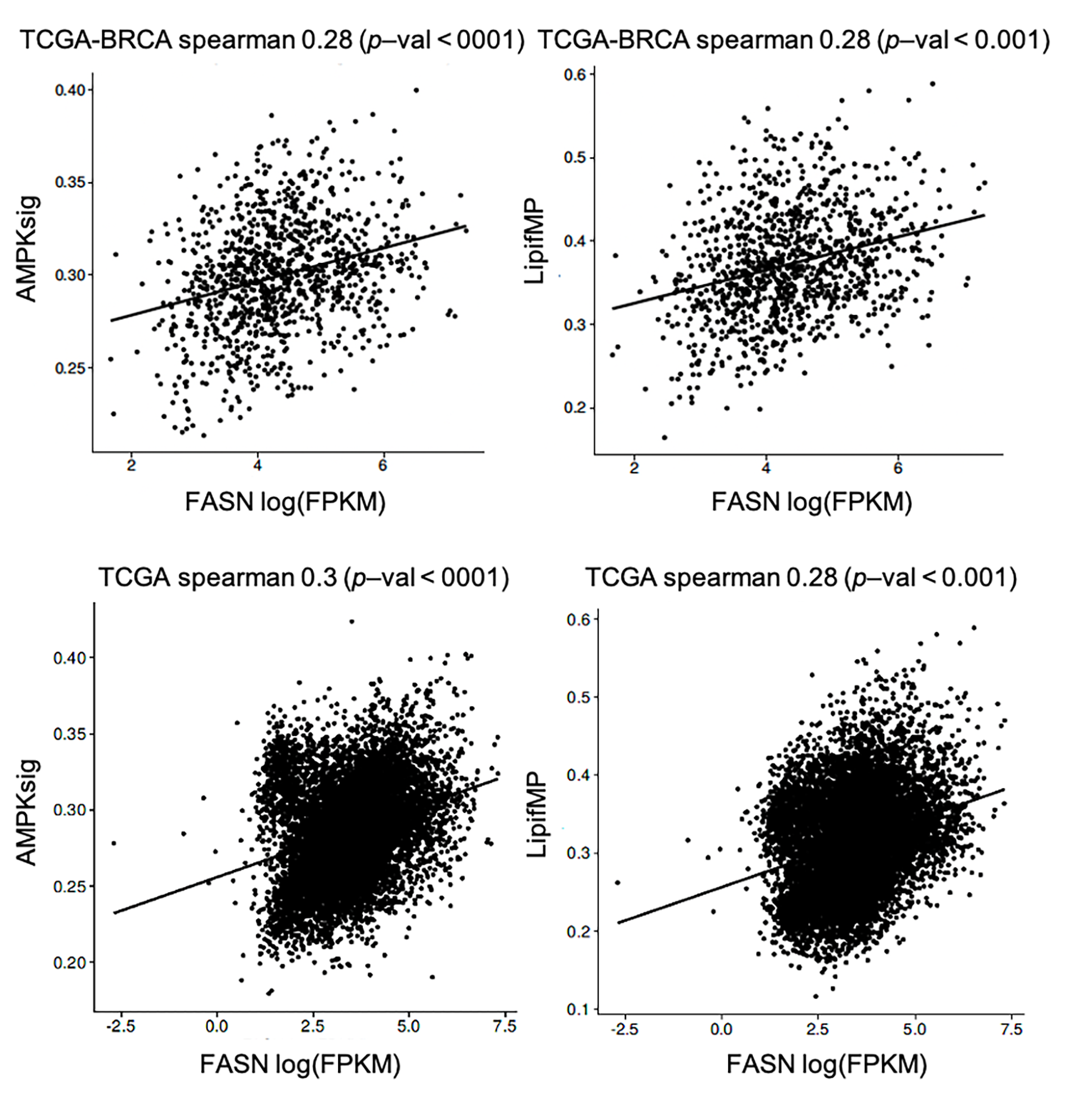
**

**Figure S2. Correlation of FASN expression with AMPK signaling and lipogenic signatures.** *Top.* Breast Cancer. *Bottom.* Pan-cancer datasets.

**Figure S3. Correlation of *FASN* expression with metabolic signatures and CRISPR-mediated gene dependencies across multiple cancer types.**

**
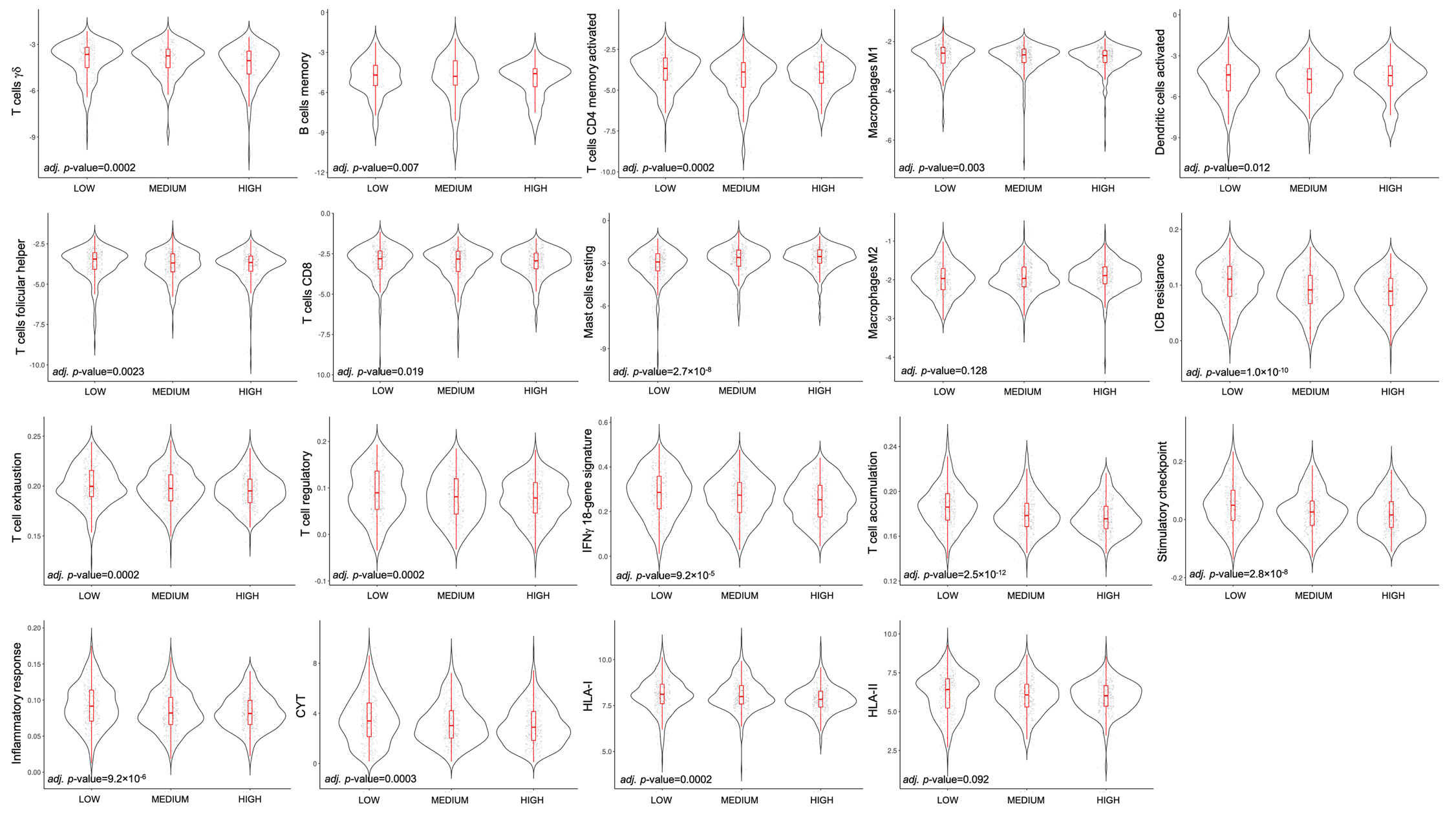
Figure S4. Differential analysis of *FASN* expression with immune cell content and immune escape signatures.** Violin plots showing adjusted ANOVA test results between *FASN* expression scores (low, medium, high) and different immune cell contents/immunotherapy response signatures.

**
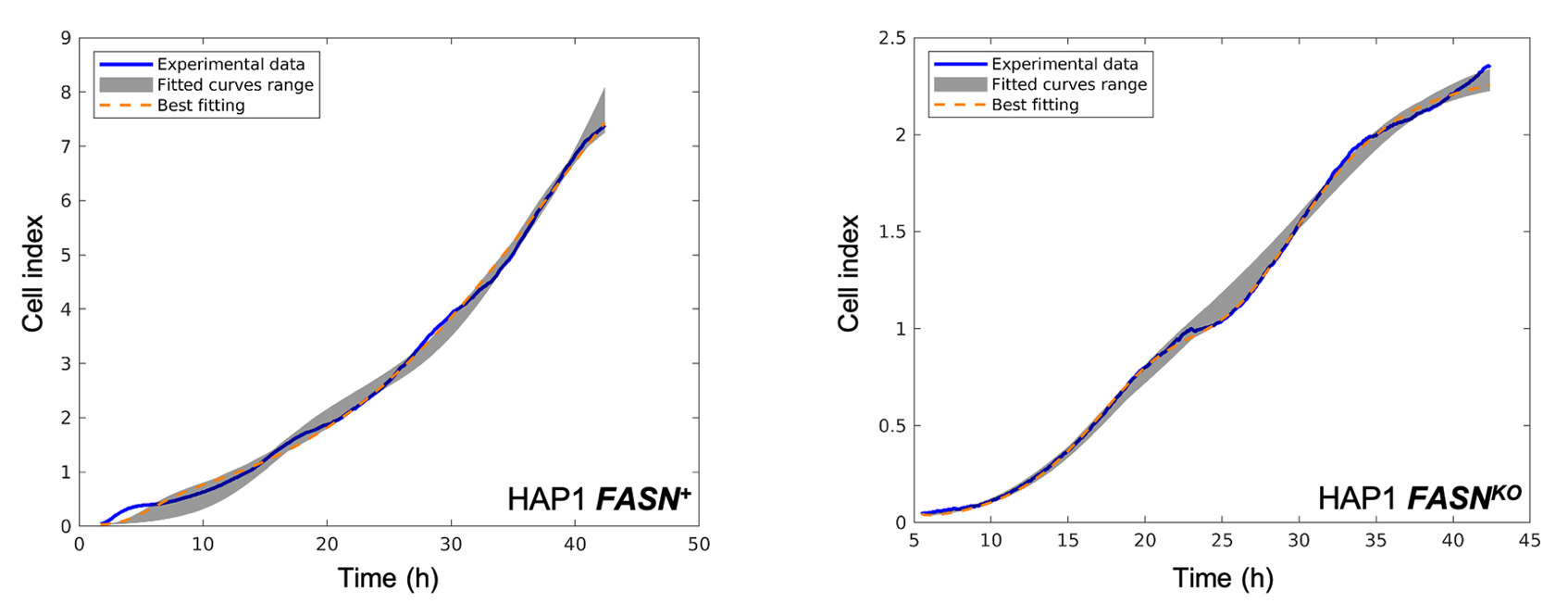
**

**Figure S5. Fitting of the mathematical model to *in vitro* cancer cell growth data.** Experimental (blue curve) impedance-based cell index (CI) data from *FASN^+^* HAP1 parental cells (*left*) and *FASN^KO^* HAP1 derivatives (*right*) growing in the absence of CATs. The orange dashed data is the best fit and the gray shading corresponds to the range of repeated fit simulations. The parameters for the best fits are: for HAP1 *FASN^+^*, r = (1.037; 7.219; 9.755), d = (5.104; 6.543; 8.737), a = (0.401; 0.791; 0.009), K = 1.129; for HAP1 *FASN^KO^*, r = (8.777; 2.979; 1.099), d = (8.430; 5.601; 0.863), a = (0.170; 0.452; 1.264), K = 13.603. The parameter values shown in triplicate correspond to the three tumor cell populations.
