# Supplementary figures and images for: Fatty acid synthase (FASN) is a tumor-cell-intrinsic metabolic checkpoint restricting T-cell immunity
Source: Cell Death Discov. 2024 Sep 30;10:417. doi: 10.1038/s41420-024-02184-z (PMC11442875; doi:10.1038/s41420-024-02184-z)

Uncropped original immunoblottings for FIG. 5B

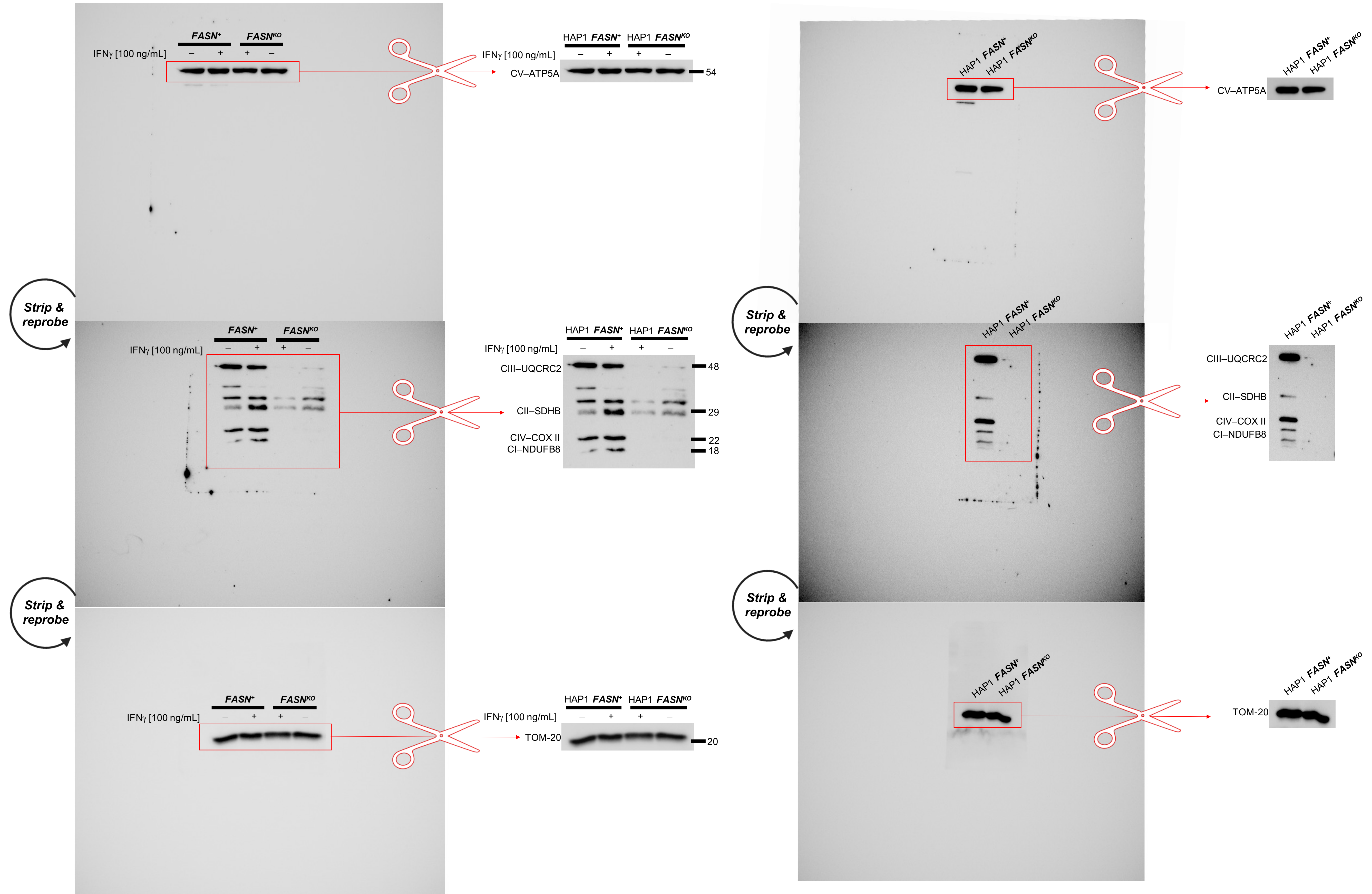

Supplement: Supplementary file 2 — Uncropped original immunoblottings [file 41420_2024_2184_MOESM2_ESM.pdf]
